# Structural and topological analysis of thiosemicarbazone-based metal complexes: computational and experimental study of bacterial biofilm inhibition and antioxidant activity

**DOI:** 10.1186/s13065-024-01338-5

**Published:** 2025-01-24

**Authors:** Doaa S. El‑Sayed, Shaymaa S. Hassan, Liblab S. Jassim, Ali Abdullah Issa, Firas AL-Oqaili, Mustafa k. Albayaty, Buthenia A. Hasoon, Majid S. Jabir, Khetam H. Rasool, Hemmat A. Elbadawy

**Affiliations:** 1 Chemistry Department, Faculty of Science, Alexandria University, Alexandria, Egypt; 2 Department of Applied Sciences, University of Technology, Baghdad, Iraq; 3 Department of Molecular and Medical Biotechnology, College of Biotechnology, Al-Nahrain University, AL-Jadriya, Baghdad, Iraq; 4 Department of Biology, College of Science, Mustansiriyah University, Baghdad, Iraq

**Keywords:** Schiff base, Thiosemicarbazide, Metal complexes, Antioxidant activity, Molecular docking

## Abstract

The structural and electronic behavior of thiosemicarbazone (TSC)-based metal complexes of Mn (II), Fe (II), and Ni (II) have been investigated. The synthesized metal complexes were characterized using elemental analysis, magnetic susceptibility, molar conductivity, FTIR, and UV–Vis spectroscopy, the computational path helped with further structural investigation. The solubility test on the TSC and its complexes revealed their solubility in most organic solvents. DFT computational analysis was performed, and quantum reactivity parameters of the octahedral optimized complexes were calculated to describe the reactivity via the stability states of the synthesized complexes. FMOs map was generated to confirm similar findings and MEP analysis was applied to elaborate the important electrophilic and nucleophilic sites on the studied surfaces. Also, other important topological analyses such as electron localization function and reduced density gradient, to establish the favorable noncovalent interactions, were studied. In silico molecular docking approach was studied against the gram-positive bacteria *Bacillus cereus* to predict the potent inhibition behavior of the studied complexes. The findings summarized the inhibition prediction of the most interactive [NiL_2_Cl_2_], then [FeL_2_Cl_2_] complexes as confirmed by the binding energy values (− 7.1 kacl/mol and − 6.4 kacl/mol, respectively). Another In silico results, with gram-positive bacteria (*S*. *aureus*), estimated similar results of the experimental finding, where [MnL_2_Cl_2_] (− 9.2 kcal/mol) is the more effective predicted antibacterial inhibitor. Fluorescence microscopy was used to examine the inhibition of bacterial biofilm, and the DPPH assay was used to measure antioxidant activity, followed by an understanding of the behavior of the current complexes toward free radicals’ removal. The findings observed less aggregated bacterial strains covered with the studied complexes leading to less dense biofilm covering.

**Supplementary Information:**

The online version contains supplementary material available at 10.1186/s13065-024-01338-5.

## Introduction

Coordination chemistry always attracted attention for its increasing importance, particularly in the design of storage facilities; slow-release or extended-release drugs in dietetics and the study of metabolism employ metal ions to accelerate drug effects. Over the years, a lot of researchers have dealt with the interaction of metal ions with DNA and its components [1]. Coordination and organometallic chemistry have extensively utilized transition metal complexes with soft or hard donor groups. Thiosemicarbazones are a class of compounds obtained by combining thiosemicarbazide with appropriate aldehydes or ketones. Thiosemicarbazones, which can bind to metals through sulfur and the hydrazine nitrogen atoms, serve as bidentate ligands in the majority of complexes; however, in certain instances, they serve as unidentifiable ligands and bond through only sulfur atoms [2–4]. Thiosemicarbazone derivatives are significant because of their numerous biological and therapeutic properties [5, 6]. Thiosemicarbazone derivatives are used in drug development to treat central nervous system disorders, bacterial infections, and analgesic and antihistamines [7]. Thiosemicarbazones, as potent intermediates in synthesizing pharmaceutical and bioactive compounds, are extensively employed in therapeutic chemistry [8]. Previous four years and assess the issues raised by the use of several sustainable solvents to extract a variety of naturally occurring bioactive chemicals. Even though some preferred solvents are regarded as environmentally friendly, we show how those solvents affect the environment and offer mitigation techniques for any potentially harmful impacts. Hashemi et al. [9] highlighted the basic physicochemical characteristics of the sustainable solvents that are typically employed to extract bioactive compounds, as well as their benefits and drawbacks, environmental issues related to those solvents, and potential future developments in this area. Regarding the use of particular green solvents in the extraction and purification of bioactive compounds, a few outstanding reviews have already been published [10–12]. DES and supercritical and subcritical solvents, for instance, are applied in the extraction of bioactive compounds from plant materials as reported by Babalo et al*.* [13]. Furthermore, thiosemicarbazones have found their way into virtually every area of chemistry; on a commercial level, they are employed in the textile, plastic, photography, and dye sectors [13]. Metal complexes interact with DNA to create novel reagents for the biological and medical sciences and have been the subject of extensive research for a considerable period [14]. Hydrolytic or oxidative pathways can be used to cleave DNA. The phosphodiester bond is cleaved during the hydrolytic process, resulting in the formation of fragments that could be discarded by an enzyme [15]. The Schiff base and transition metal have not been worked on, according to a search through the literature. Thiosemicarbazone compounds have shown a wide spectrum of biological activity in the fields of antimicrobial [16], antitumor [17], sodium channel blocker [18], anticancer [19], antitubercular, and corrosion inhibitor [20]. Considering the diverse biomedical uses of this class of compounds, we present the synthesis and investigation of Fe (II), Mn(II), and Ni(II) complexes of thiosemicarbazide derivatives. The metal synthesized octahedral complexes based on thiosemicarbazone were predicted to have a potent therapeutic strategy towards pharmaceutical field. Several candidates were studied with four and five coordinate structures [21] and others showed a broad variety of coordination numbers and geometries, different oxidized and reduced states, and the intrinsic ligand characteristics provide chemists studying pharmaceuticals with an abundance of drug structures [22].

**Scheme 1:**
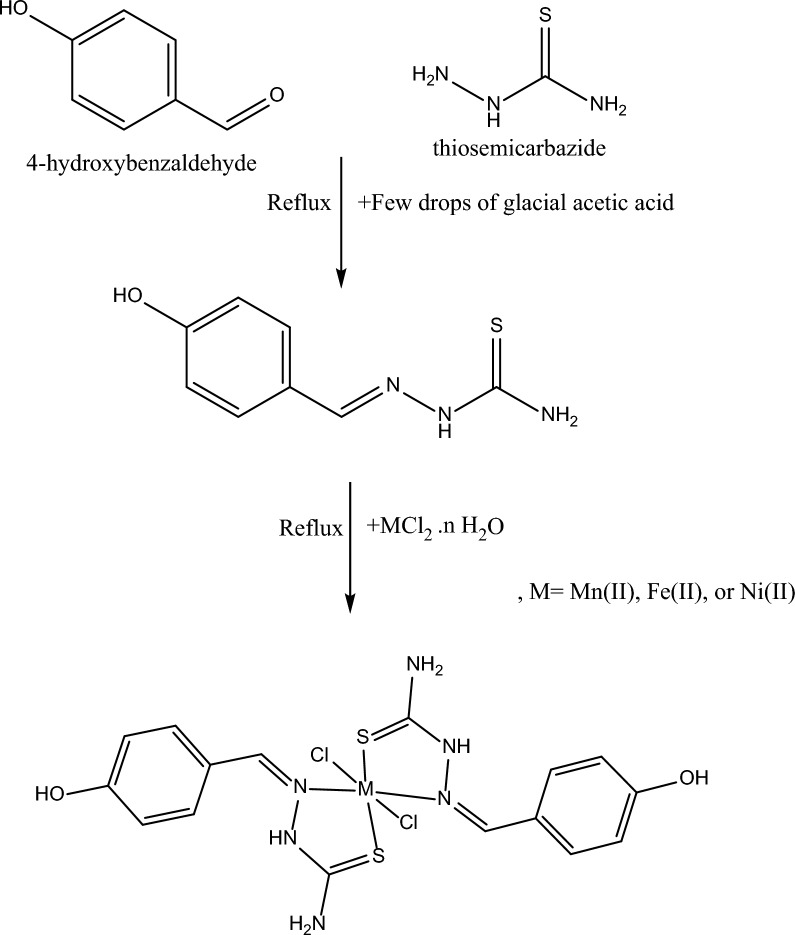
Synthesis of the Schiff base (TSC) and its complexes

The aim of this study is to synthesize thiosemicarbazone (TSC)-based metal complexes as sketched in Scheme 1. Using IR and UV spectra, the molecular structures of TSC-M complexes were ascertained (MS). As the synthesis and characterization of these complexes were achieved previously in the literature [21, 23], we directed to investigate their electronic structures computationally in more advance. Important quantum parameters for the optimized structures were calculated based on FMOs. In this work, topological analysis was considered to give a full description of the coordinate nature between donor and acceptor terminals, such as MEP, ELF, and RDG/NCI indices. DPPH assay was used to determine the antioxidant activity to realize the behavior of the current complexes toward free radicals’ removal. The best ligand–protein interactive score was determined by protein macromolecular analysis using the tested complexes.

## Materials and methods

This work employed reagent-grade chemicals from the Fluka company and Sigma-Aldrich, including MnCl_2_.4H_2_O (98% Fluka), FeCl_2_.4H_2_O (≥ 99.0% Fluka), and NiCl_2_.6H_2_O (≥ 98% Fluka), Thiosimcarbazide, 4-Hydroxybenzaldehyde (98% Sigma-Aldrich), and absolute ethanol (≥ 99.8% Fluka). On a Shimadzu 3800- FTIR spectrophotometer, CsI discs, Fourier Transfer Infrared Rays (FTIR) spectra were recorded in the range (4000–400) cm^−1^. Electronic spectra of products were recorded in the range of 200–1100 nm using a Shimadzu UV-1650 spectrophotometer, in freshly prepared 10^–3^ M ethanolic solutions. A Shimadzu A680G was used to measure the metals in the complexes-atomic absorption spectrophotometer. A capacitor analyzer was used to measure the conductivity of the complexes at 25 °C in a freshly prepared 10^–3^ M solution in Ethanol. The balanced magnetic susceptibility of Bruke magnet B.M.6, England, was used to collect magnetic properties. The melting points of the prepared compounds were determined using the Gallenkamp M.F.B-600 F Melting Point Apparatus.

### Synthesis of (E)-2-(4-hydroxybenzylidene)hydrazine-1-carbothioamide (Schiff base- TSC)

20 mL of ethanolic solution of 4- Hydroxybenzaldehyde (0.122 g, 1 mol) was refluxed with 20 mL of thiosemicarbazide (0.091 g, 1 mol), and a few drops of glacial Acetic acid were added. The reflux was applied for 6 h, then allowed to cool at room temperature. The produced black precipitate was filtered and recrystallized from ethanol to give black needles [24].

### Synthesis of dichloro-bis(2-(4-hydroxybenzylidene)hydrazine-1-carbothioamido) metal(II) complexes:

20 mL Ethanolic solution of ligand (L) (0.400 g, 2 mol) was added to an aqueous solution of each of the metal salts (1 mol) (0.198 g MnCl_2_.4H_2_O, 0.199 g FeCl_2_.4H_2_O, and 0.238 g NiCl_2_.6H_2_O). The reaction mixture was continuously stirred for 2 h. The required product was shortly precipitated at room temperature, filtered off, and washed with 1:1 (ethanol: water), recrystallized from ethanol, and dried at 70 °C. Scheme 1 displays the synthesis of the Schiff base ligand (L) and its metal complexes.

### Computational details:

To suggest a potential 3D structure for the studied Fe, Ni, and Mn complexes, density functional theory (DFT) geometrical optimization, that performed for theoretical structure investigation, and calculations were performed in the gas phase [25–27]. The exchange–correlation functional B3LYP with GENECP basis set was applied to involve 6-31g for the atoms C, H, N, O, S, and Cl, and LANL2DZ, the computational basis set that treat the core potential of transition elements, was utilized for the metal atoms Fe, Ni, and Mn. The computations were performed using the Gaussian 09 W and Gaussian View software [28]. FMOs were described based on chk file geometrical generation. The quantum chemical reactivity parameters were estimated for all studied complexes after geometrical optimization. The following Eqs. [29, 30] were used to determine these descriptors using the values of HOMO, LUMO, the ionization potential (I), and the electron affinity (A):

1 $${\text{Energy gap }}\left( {{\text{E}}_{{{\text{GAP}}}} } \right) \, = {\text{ E}}_{{{\text{LUMO}}}} - {\text{ E}}_{{{\text{HOMO}}}}$$

2 $${\text{Ionization potential }}\left( {\text{I}} \right) \, = \, - {\text{ E}}_{{{\text{HOMO}}}}$$

3 $${\text{Electron affinity }}\left( {\text{A}} \right) \, = \, - {\text{ E}}_{{{\text{LUMO}}}}$$

4 $${\text{Chemical hardness }}\left( \eta \right) \, = \, \left( {{\text{I}} - {\text{A}}} \right)/{2}$$

5 $${\text{Chemical potential }}\left( \mu \right) \, = \, - \, \left( {{\text{I}} + {\text{A}}} \right)/{2}$$

6 $${\text{Softness }}\left( \sigma \right) \, = { 1}/ \, \eta$$

7 $${\text{Electronegativity }}\left( \chi \right) \, = \, - \left( {{\text{E}}_{{{\text{HOMO}}}} + {\text{ E}}_{{{\text{LUMO}}}} } \right)/{2}$$

8 $${\text{Electrophilicity }}\left( \omega \right) \, = \, \mu^{{2}} /{2}\eta$$

### Molecular docking methodology

For very effective outcomes, the docking process primarily follows a critical path [31–33]. Consequently, the Autodock Vina [34] software (version 4.2) was used to simulate the docking of the studied complexes with control comparison. Moreover, analysis and visualization of docking data were performed using Discovery Studio software (https://www.3ds.com/products-services/biovia/). Transferase enzyme of gram-positive *Bacillus cereus* bacteria (ID: 4JH9) [35] was selected, based on the biological potent evidence of the inhibition effect. The protein of interest and was obtained from the Protein Data Bank website (https://www.rcsb.org/structure/4JH9). The optimized complexes were docked under certain conditions starting with preparing the target protein by removing water molecules and any undesirable atoms. The protein charge was adjusted after adding polar H-atoms, and the complexes were applied as pdbqt extension files. The expected active spots were identified, and the grid box dimensions were created [36, 37]. With an energy range of 4, the grid box size was approximated based on the drug-like control inside the protein pocket, with dimensions of 40 × 40 × 40 Å, 0.3 Å spacing, grid centers x, y, and z of -2.343, 2.477, and 5.872, respectively. Lamarckian-Genetic Algorithm was thought to be the binding affinity mode [38–40].

### Bacteria pathogenic identification and diagnosis

Isolates of (*B. cereus, and K. pneumoniae*) were supplied from the laboratory of microbiology. All microbial isolates were identified at the species level by the VITEK-2 compact system. This can be processed with a (GP) card for gram-positive bacteria identification, and a (GN) card for gram-negative bacteria identification.

### Estimation of minimum inhibitory concentrations (MIC)

The Minimum Inhibitory Concentration (MIC) of FeL_2_Cl_2_, NiL_2_Cl_2_, and MnL_2_Cl_2_ against *B. cereus* and *K.pneumoniae* was determined using a microdilution assay. The procedure involved the following steps: Stock solutions of FeL_2_Cl_2_, NiL_2_Cl_2_, and MnL_2_Cl_2_ were prepared at various concentrations ranging from 1024 to 1 µgmL^-1^. Test microliter plates were set up with a twofold dilution series for each treatment group, including FeL_2_Cl_2_, NiL_2_Cl_2_, and MnL_2_Cl_2_. Each well of the plates was inoculated with a standardized suspension of *B. cereus* or/and *K.pneumoniae* at a concentration appropriate for the assay. The plates were incubated at 37 °C for 24 h to allow for bacterial growth and interaction with the treatments. After incubation, the MIC was determined as the lowest concentration of the agent that inhibited visible bacterial growth.

### Antimicrobial assay

The antibacterial activity was examined using the Agar well diffusion method [41] according to the manufacturer’s instruction. Mueller Hinton agar was prepared and used, 0.1 ml of overnight strain culture (adjusted to 0.5 McFarland turbidity), streaked entirely on the Mueller Hinton agar using a sterile swab stick. Four wells were bored in the culture medium using a sterile 6 mm diameter cork borer. 0.1 ml of each concentration (25,50,100) µg/ml of metal complexes: ([FeL_2_Cl_2_], [NiL_2_Cl_2_], [MnL_2_Cl_2_] were added to different wells using a sterile micropipette. The negative control had D.W. The observed inhibition zones were measured and recorded in millimeters. This was done in triplicates to study biofilms formed on culture dishes of Lysogenia broth medium (HiMedia, India). Bacterial strains were left unstained as control or treated with ([FeL_2_Cl_2_], [NiL_2_Cl_2_], and [MnL_2_Cl_2_]) at a concentration of 100 μg/mL for 24 h and stained using the Film Tracer LIVE/ DEAD Biofilm Viability Kit. The results were examined using a fluorescent microscope [42].

### Antioxidant activity by DPPH

Using the DPPH (2,2-diphenyl-1-picryl-hydrazyl-hydrate) radical, scavenging activities of the products evolved, based on the methodology described in the previous work [43]. 10 µL of each component ([FeL_2_Cl_2_], [NiL_2_Cl_2_], [MnL_2_Cl_2_]) of concentrations; 25, 50, and 100 µg/mL 490 µL was added to the DPPH in absolute ethanol. The samples were incubated for 30 min at 25 °C, and the absorbances were measured at 517 nm. A DPPH solution only (500 µL) was prepared as a blank solution. The measurements were carried out three times. According to Eq. (9), the antioxidant activity was determined [44].

9 $$\text{Scavenging activity \% }=\frac{{OD}^{control}-{OD}^{sample}}{{OD}^{control}}x100$$

### Statistical analysis

The data of the current study were statically analyzed by an unpaired t-test using GraphPad Prism (version 7) (8). The values were represented as the mean ± SD [45].

## Results and discussion

The reactions of Mn, Fe, and Ni(II) chloride salts with synthesized TSC-Schiff base (L) in 1:2 molar ratios were followed, and Physiochemical characterizations of the products were investigated. The products are found to be air-stable, at room temperature. The TSC- Schiff base (L) is soluble in common organic solvents, such as ethanol, methanol, and chloroform, while the metal complexes are relatively well soluble in Ethanol. The synthesized ligand and its complexes were characterized by elemental analysis, UV spectra, FTIR, and conductivity measurements. The physical properties and data of the ligand (HL) with its metal complexes are given in Table 1. The small values of conductivity measurements indicated that metal complexes are non-ionized, while the metal analysis suggested the mole ratio of metal complexes to be 1 M(II): 2L: 2Cl.

**Table 1:** Physical characteristics and analytical data for (TSC) and its metal complexes

Compound	Colour	Melting Point (^o^C)	Yield %	Conductivity (μs.cm^−1^)	Elemental analysis Calc. (found)				
					C	H	N	S	M
Ligand (L)	Black	185–187	80	–	49.22 (49.00)	4.65 (4.52)	21.52 (22.02)	16.42 (16.02)	–
[FeL_2_Cl_2_]	Brown	197–199	80	49	37.16 (38.02)	3.51 (3.82)	16.25 (16.62)	12.40 (12.66)	10.80 (10.49)
[MnL_2_Cl_2_]	Light pink	204–206	76	54	37.22 (37.50)	3.51 (3.42)	16.28 (16.80)	12.42 (12.86)	10.64 (10.21)
[NiL_2_Cl_2_]	Dark Green	200–202	77	44	36.95 (36.81)	3.49 (3.31)	16.16 (15.99)	12.33 (12.01)	11.29 (11.22)

### Infrared spectra

**Fig. 1:**
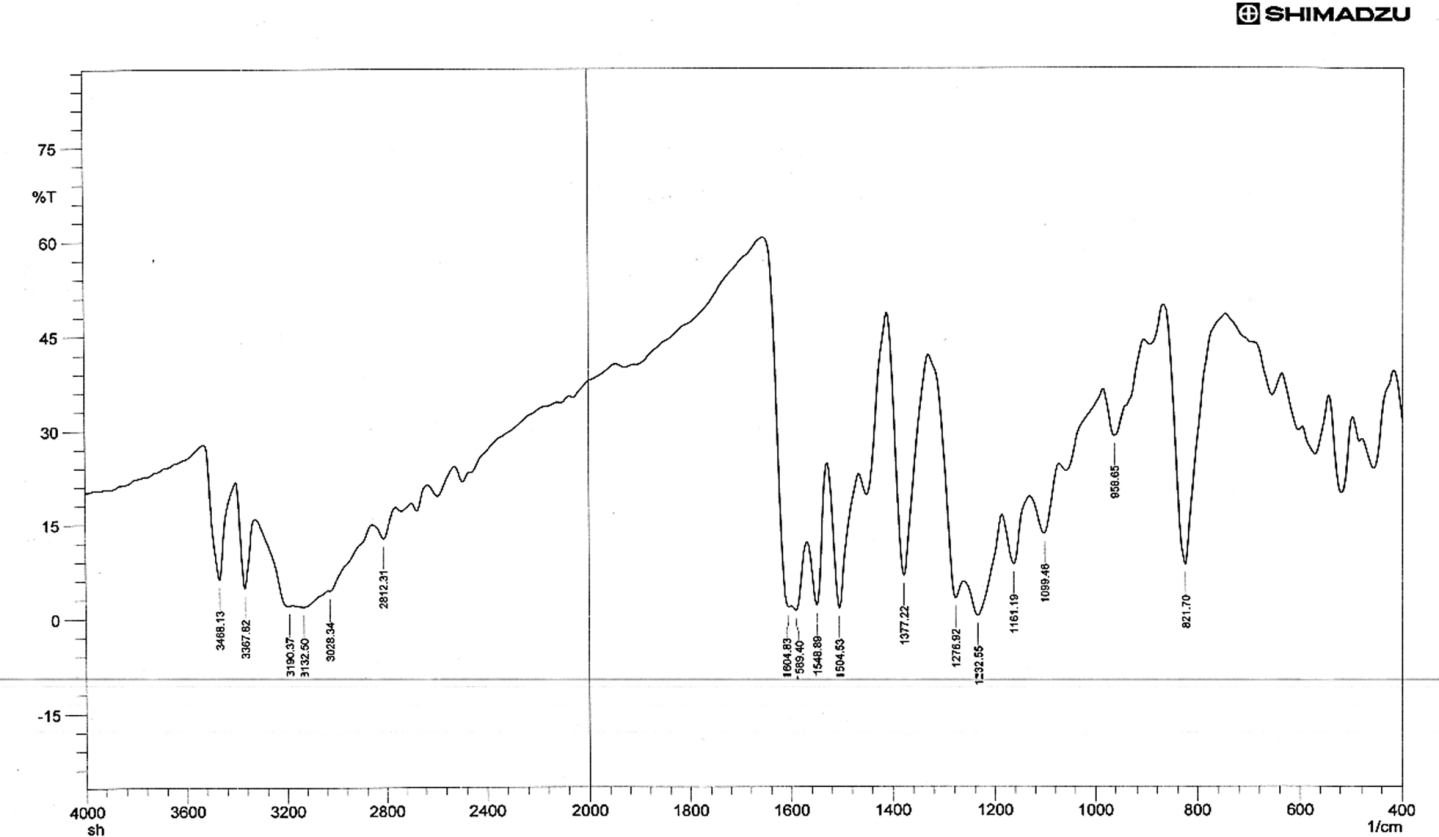
FT-IR spectrum of Schiff base, L

The type of functional group that is affixed to the metal atom can be inferred from the IR spectra, which offer important insights. The FTIR spectra of the ligand TSC (Fig. 1) showed strong bands in the 3265, 3157 cm^–1^ assignable attributed to the − NH_2_ group and the strong bands in the 3421 cm^−1^ assignable to the − NH group. The appearance of these peaks in all the spectra of the complexes indicates that the –NH_2_ and − NH groups are not involved in complexation [46–48].

**Fig. 2:**
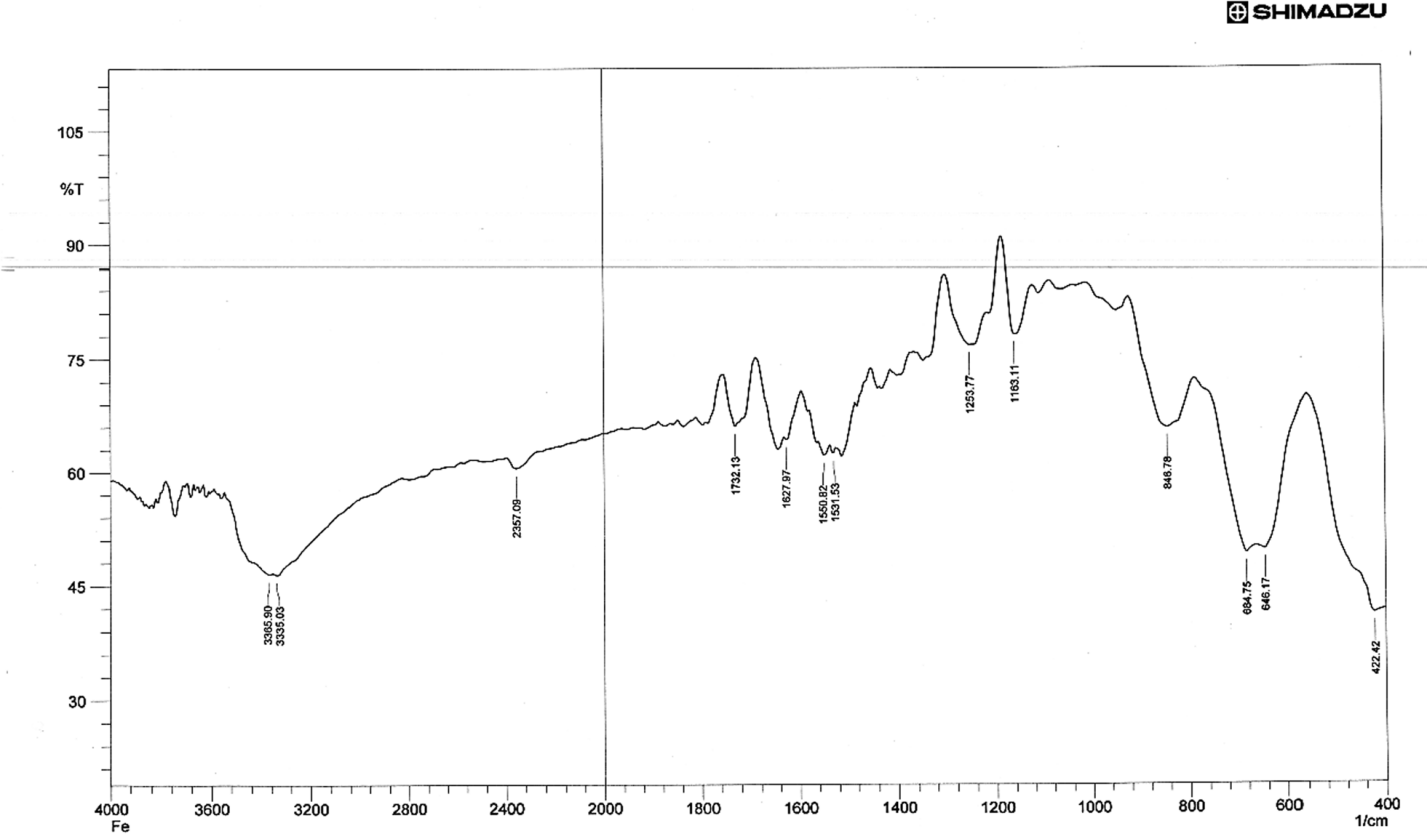
FT-IR spectrum of compound (FeL)

**Fig. 3:**
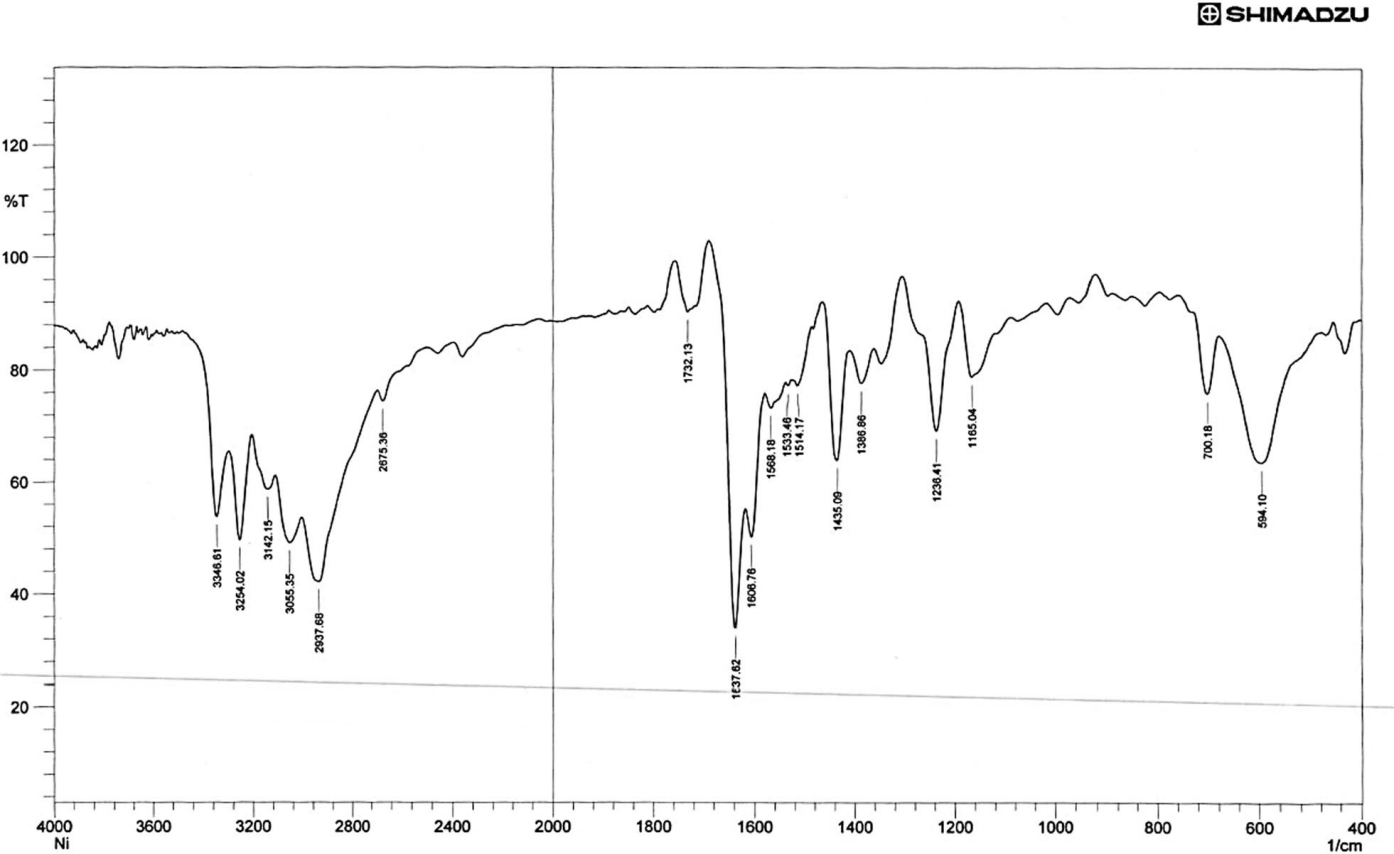
FT-IR spectrum of compound (NiL)

**Fig. 4:**
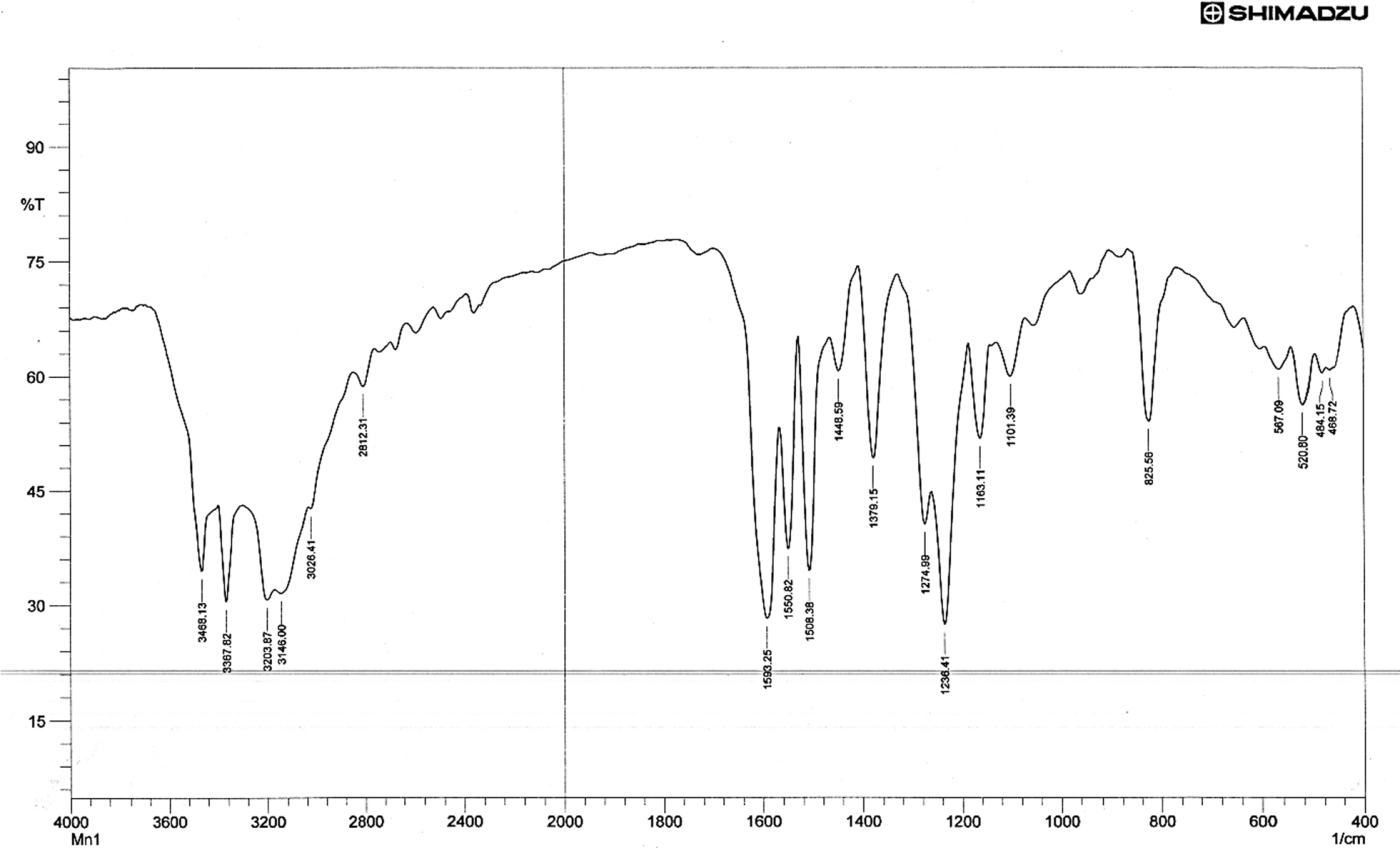
FT-IR spectrum of compound (MnL)

A strong absorption band at 1583 cm^−1^ due to ν (C = N) present in the free ligand has red shifted by 15–25 cm^−1^ in all complexes, indicating the involvement of (C = N) azo methine group in coordination [49], The strong bands observed at 1278 cm^−1^ & 831 cm^–1^ in the spectrum was due to the ν(C = S) and δ(C = S) [50]. The band near (1165) cm^−1^ in the free Schiff base ligand may be assigned to υ (NH–C = S), which showed a red shift upon complexation indicating the sulfur contribution in coordination with the metal ion [51]. Accordingly, the ligand acts as a bidentate chelating agent, bonded to the metal ion via the nitrogen (–C = N) atoms and the Sulfur (–C = S) atoms of the Schiff base ligand for the Ni(II), Mn(II), and Fe(II) complexes. The far IR spectra of the metal chelate show some new bands at 543 ± 10 cm^−1^ and 501 ± 10 cm^−1^ have been assigned to ν (M–N) and ν (M–S) modes respectively [52, 53], Figs. 2, 3, 4. Table 2 displays the FTIR data exported for both TSC and its metal complexes. However, the stretching vibration of C = N was shown to be equal to 1623, 1605, and 1624 cm^−1^ in the Fe, Mn, and Ni complexes respectively. The ν_(C=S)_ were found to be 792 and 678 cm^−1^ for the Fe complex, 753 and 690 cm^−1^ for the Mn complex, 758 and 688 cm^−1^ for the Ni complex. These observed shifts can be due to changes in bond strength and coordination environment caused by the coordination of the mentioned functional groups with metal ions. The band near (1165) cm^−1^ in the free Schiff base ligand assigned to the stretching vibration of (NH–C = S) showed a shift to lower wave numbers in the corresponding complexes (1050 cm^−1^ for Fe complex, 1059 cm^−1^ for Mn complex and 1050 cm^−1^ for Ni complex) confirming sulfur contribution in coordinating to the metal ion. Additionally, the phenolic OH group appeared at 2367 cm^−1^ in ligand seems not very much affected upon metal complexation showing little shift that can be attributed to total environment.

**Table 2:** The most diagnostic FTIR peaks for the ligand, L and its complexes (cm^−1^)

Compounds	**υ**_**NH2**_	**υ**_**NH**_	**υ **_**(NC=S)**_	**ν**_**(C=N)**_	δ _**(C=S-)**_	**ν**_**(M-S)**_	**ν**_**(M–N)**_
L	3265 3157	3421	1165	1583	831	–	–
[FeL_2_Cl_2_]	3250 3150	3388	1050	1623	792 678	307	420
[MnL_2_Cl_2_]	3248 3153	3384	1059	1605	753 690	311	435
[NiL_2_Cl_2_]	3245 3156	3382	1050	1624	758 688	305	435

### Electronic spectra

The UV–Vis electronic spectra of thiosemicarbazide Schiff base metal complexes reveal important information about their electronic structure, coordination geometry, and metal–ligand interactions [54–59].

**Fig. 5:**
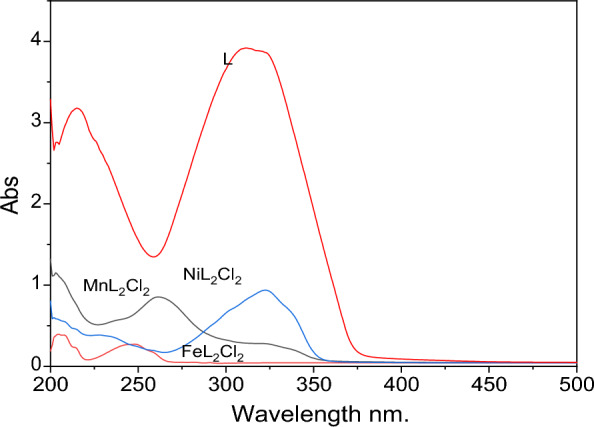
Electronic Spectra of compounds (L, NiL_2_Cl_2_, FeL_2_Cl_2_, and MnL_2_Cl_2_)

The electronic spectra of the ligands and the Ni (II), Mn (II), and Fe (II) complexes were recorded in Ethanol at room temperature (Fig. 5). The UV spectral data of free ligand L shows a strong band at 322 nm which may be attributed to the benzene n–π* transition and another at 215, 240, and 311 nm due to π–π* transition of the nonbonding electrons azomethine nitrogen in the Schiff base. The UV spectra of the studied ligand complexes show a significant variation in spectral lines at a range from 200 nm to 400 nm.

### Magnetic properties

The magnetic moment *μ*_*eff*_ for the complexes of Fe^2+^ (d^6^), and Mn^2+^ (d^5^) was found to be 5.47 B.M and 6.40 B.M. respectively, which were within the expected spin-only values^.^ The higher value of *μ*_*eff*_ of the Ni^2+^ (d^8^) complex 3.21 B. M can be attributed to the orbital contribution. Finally, the values of the magnetic moment of the complexes confirm the octahedral geometry of the prepared complexes [60, 61]. According to the magnetic moment values, all the prepared complexes are paramagnetic, and their values do not deviate from the theoretically calculated ones, they also agree with those reported for typical transition metal complexes. These results are found in Table 3.

**Table 3:** The magnetic properties of the complexes at 25 °C

Complexes	No. of Elec.	No. of unpaired electron	Electronic configuration	Term symbol	*μ*_*eff*_	
					Calc.	Found
[MnL_2_Cl_2_]	d^5^	5	T_2_g^3^ Eg^2^	^6^S	5.92	6.40
[FeL_2_Cl_2_]	d^6^	4	T_2_g^4^Eg^2^	^5^D	4.90	5.47
[NiL_2_Cl_2_]	d^8^	2	T_2_g^6^Eg^2^	^3^F	3.87	3.21

### Geometrical achievement

**Fig. 6:**
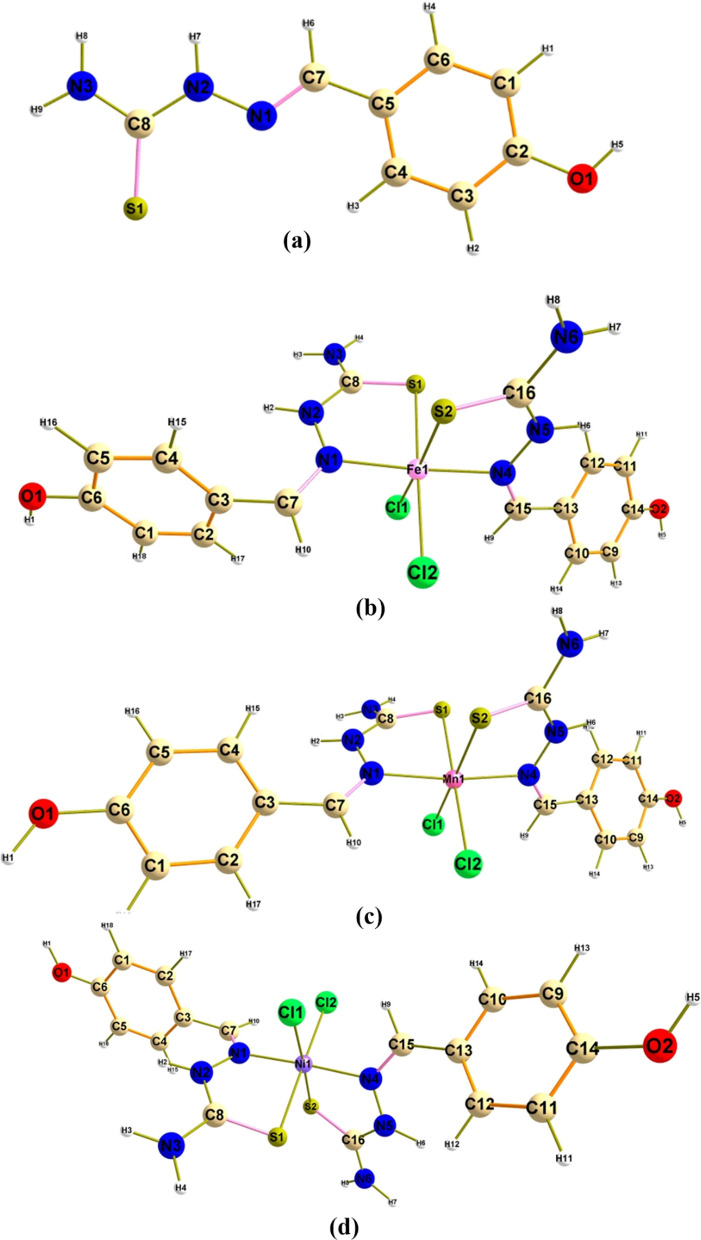
Geometrical optimized complexes. **a** Schiff base (TSC), **b** Fe(II), **c** Mn(II) and **d** Ni(II), using DFT/B3LYP/GENECP level (atomic color scale, S, yellow; N, blue; O, red; C, grey; H, white)

The designed complexes under investigation have optimal geometries displayed in Fig. 6 the electronic system mainly depends on the relationship between the equilibrium geometries of the studied complexes and the electronic structure of the transition metal atoms. Geometrically optimized structures described the octahedral geometry of all metals coordinated with two bidentate ligand molecules from its S and N binding sites, and two chloride atoms leading to balance the charge of the metal ion. Considering the bond length variation between the ligand atoms before and after complexation (Supplementary Figs. 1–4) can detect this change. Cartesian coordinates of the designed complexes are displayed in Supplementary Tables 1–4.

### Quantum chemical reactivity and FMOs analysis

**Fig. 7:**
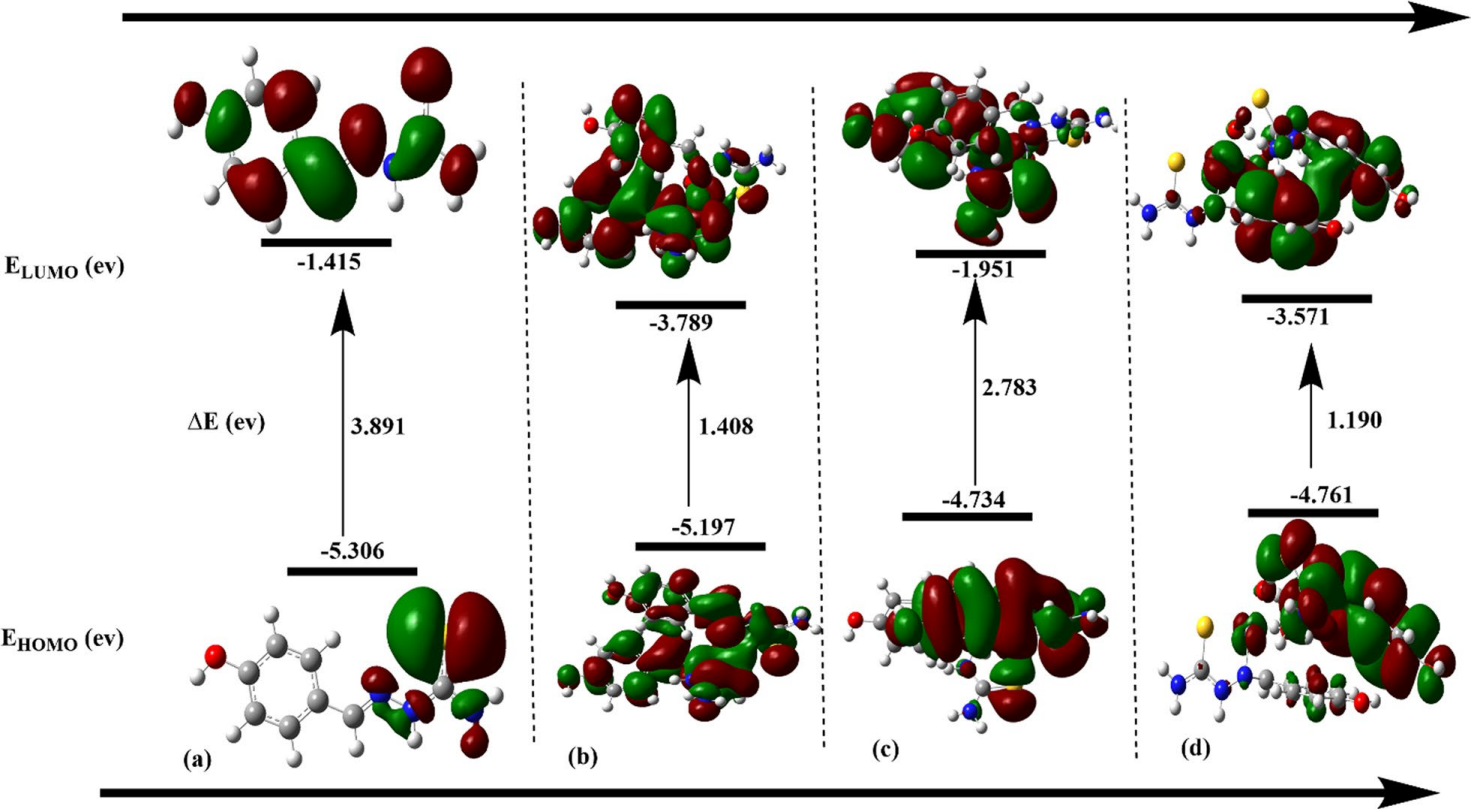
Energy levels of FMOs for the studied complexes, **a** Schiff base (TSC), **b** Fe(II), **c** Mn(II), **d** Ni(II)

The highest occupied and lowest unoccupied molecular orbitals (HOMO and LUMO) were generated for all complexes describing different electronic contributions during electronic transitions. Differences in FMO energies were mentioned as the energy gap values that indicate the stability level of the studied complexes. From Fig. 7, it is obvious that Fe(II) complex is the most stable as its energy gap is estimated at 1.252 eV and the LUMO energy has a slightly high estimated value (− 4.952 eV). On the other hand, Ni(II) complex was considered the most reactive system during electronic transitions (1.011 eV), and its transition analysis is closer to Mn(II) complex (1.07 eV). Using the DFT method with B3LYP/GENECP level, some reactivity descriptors were calculated to understand their aspects in therapeutic and toxicology. The most important descriptors included the global electrophilicity index (ω), hardness (η), softness (σ), electronegativity (χ), chemical potential (μ), and softness (σ) are listed in Table 4**.** Because there are differences in electron mobility between LUMO and HOMO, the energy gap (∆E) is essential for ranking reactivity [62]. A molecule with a smaller ∆E is more reactive. As a result, the order of reactivity should be [Ni(TSC)_2_Cl_2_] > [Mn(TSC)_2_Cl_2_] > [Fe(TSC)_2_Cl_2_]. The reactivity and electron density fluctuation rate of a molecule can be used to measure how “soft” it is [63]. This shows that charges can be transferred between the relevant compounds quite simply. The hardness and softness of molecules should have opposite strengths in order to properly arrange reactivity [64]. This is because a molecule that is more reactive would be softer and less hard than a molecule that is less reactive. As in [Ni(TSC)_2_Cl_2_] that is more reactive due to its higher softness (1.976).

**Table 4:** Quantum chemical reactivity parameters of the studied complexes

complex	E_HOMO_	E_LUMO_	E_GAP_	I	A	μ	χ	ω	η	σ
Schiff base (TSC)	− 5.306	− 1.415	3.891	5.306	1.415	3.360-	3.360	2.902	1.945	0.514
[Fe(TSC)_2_Cl_2_]	− 6.204	− 4.952	1.252	6.204	4.952	− 5.578	5.578	24.851	0.626	1.597
[Mn(TSC)_2_Cl_2_]	− 6.195	− 5.125	1.07	6.195	5.125	− 5.66	5.66	29.94	0.535	1.869
[Ni(TSC)_2_Cl_2_]	− 5.990	− 4.979	1.011	5.990	4.979	− 5.485	5.485	29.728	0.506	1.976

The resistance of molecules to electron density deformation under minor perturbations during chemical reactions can be determined by analyzing their hardness and softness indices. Hard molecules have a wide HOMO–LUMO gap and are not very polarizable, whereas soft molecules have a short HOMO–LUMO gap, are highly polarizable, and are more reactive [65]. An important indicator of global chemical reactivity is the global electrophilicity index, which quantifies how energy stabilizes when a molecule receives an additional electronic charge from an outside source [66]. According to DFT calculations, there is a strong interaction between the studied ligand and Fe(II), Mn(II), and Ni(II) ions through the nitrogen and sulfur atoms of the designed ligand. Furthermore, the determined energy gap between FMOs demonstrated that the produced complexes were sufficiently stable to be synthesized and employed successfully for pharmacological application. Table 4 illustrates that all of the complexes' chemical potential values are negative, signifying their stability and resistance to dissociating back into their constituent materials following production. Furthermore, the data demonstrated that the hardness (η) values of the proposed complexes were small with the greater softness (σ) values, indicating that the studied complexes possess a small energy gap leading to reactive scale prediction.

### Topological interpretation

#### Molecular electrostatic potential (MEP)

The distribution of charges, the locations of electrophilic and nucleophilic assaults inside the molecule, and molecular reactivity are all clarified by molecular electrostatic potential [67]. The visualized colored map can be generated utilizing a specific force acting on specific species in the molecule generating a predicted potential depending on the electron cloud and nuclei at a certain point. MEP is expressed by the following equation:

$${\varvec{V}}\left({\varvec{r}}\right)=\sum_{{\varvec{A}}}^{{\varvec{N}}}\frac{{{\varvec{Z}}}_{{\varvec{A}}\boldsymbol{ }}}{{\varvec{r}}-{{\varvec{R}}}_{{\varvec{A}}}}-{\varvec{\rho}}\left({{\varvec{r}}}^{\boldsymbol{^{\prime}}}\right){{\varvec{d}}}^{3}{\varvec{r}}\boldsymbol{^{\prime}}({\varvec{r}}-{{\varvec{r}}}^{\boldsymbol{^{\prime}}})$$

**Fig. 8:**
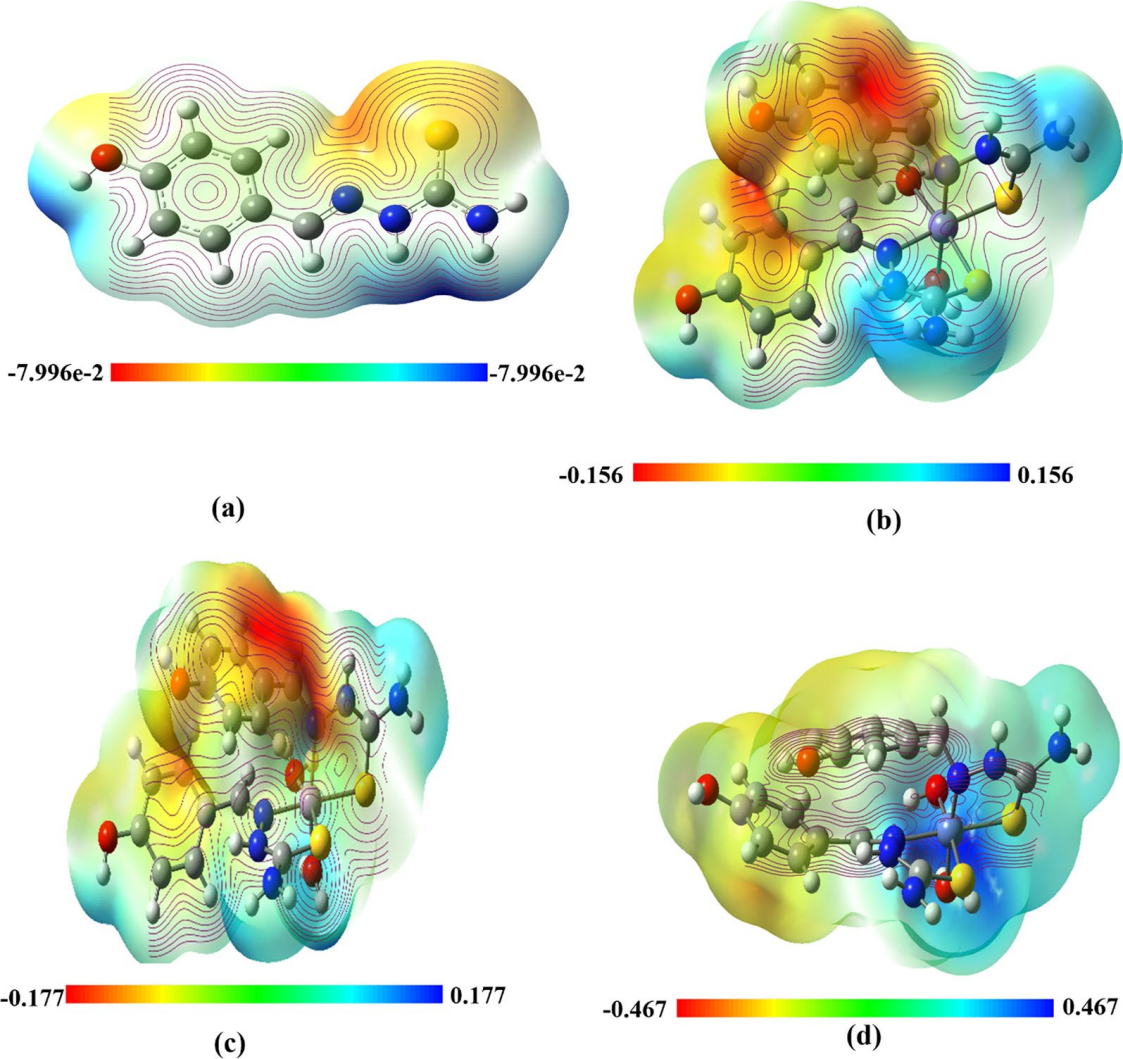
3D-MEP surface of the studied complexes, **a** Schiff base (TSC), **b** [Fe(TSC)_2_(Cl)_2_], **c** [Mn(TSC)_2_(Cl)_2_], and **d** [Ni(TSC)_2_(Cl)_2_], (red and yellow color scales refer to electron-rich sites, pale and dark blue color scale refer to electron-poor sites)

In this work, the MEP 3D colored map of M(II) complexes was exported in Fig. 8 significant increase in potential pass-through red color scale (more negative value) to blue color scale (more positive value). Red denotes the largest negative electrostatic potential region, whereas blue denotes a positive electrostatic potential [68]. It was observed that the electron density around S atom of the ligand is varied after complexation with the studied M(II) ions. That predicted more electron transfer to the metal ion through the donor S atom. In more detail, it was observed from the mapped results that the highly electronic-rich sites present in the aromatic rings of the ligands for both Fe(II) and Mn(II) complexes. However this contribution significantly less appeared in Ni(II) complex. In all complexes, the electronic poor sites referred to with the blue color scale delocalized on the NH_2_ group of the ligand leading to think about NH_2_ group sharing in other reactions such as intramolecular hydrogen sulfur atom of the ligand.

#### Electron localization function (ELF)

**Fig. 9:**
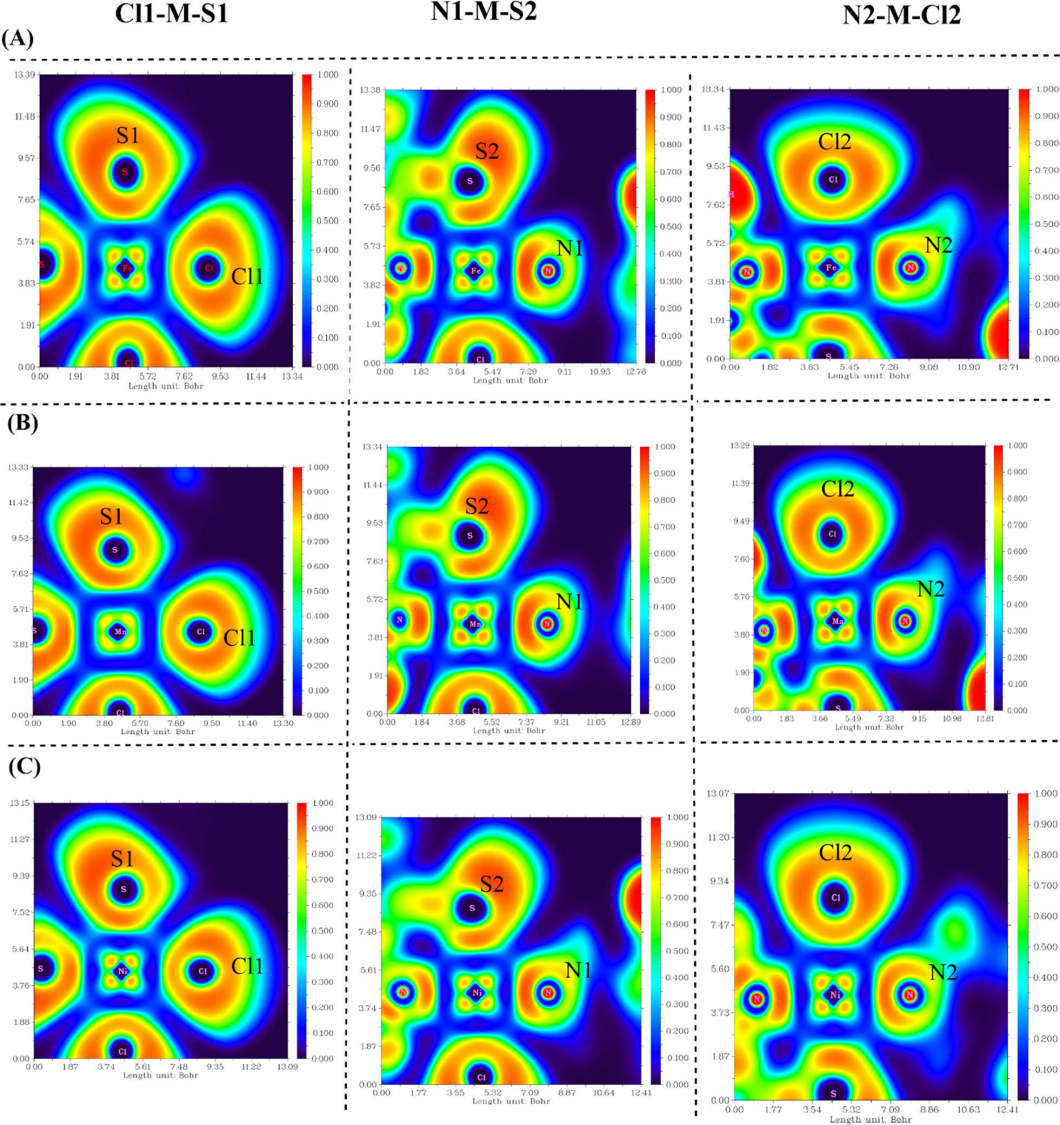
ELF map involving several atomic planes in M-TSC complexes, **A** Fe(II), **B** Mn(II), and **C** Ni(II), with three atomic planes Cl1-M-S1, N1-M-S2, N2-M-Cl2, (red color scale refers to electron localization areas)

The identification and prediction of electron localization zones on the surface of the coordinated metal complexes can be facilitated by ELF. For the studied M-TSC complexes, the 2D shaded surface map is displayed in Fig. 9. The plane is characterized by three atoms involving the core metal and coordinated two donor atoms of TSC ligand. The map illustrates the difference in electron localization density surrounding this three-atom plane, denoted by a distinctive color code [69]. In the case of Fe(II) complex, the three planes described the nature of the coordinate bond based on the strength of the donor site to share its electrons, as in the case of Cl1–M–S1 plane with significant delocalized electrons distributed around the Fe-coordinated sites, unlike N2, N1 and S2 atoms in the other described planes. On the other hand, Mn planes were best described for strong interactions through N1–Mn–S2 and N2–Mn–Cl2 planes, while Mn with Cl1 and S1 exhibited low electronic area around the metal. In the case of Ni-complex, the same finding is present as in Fe-complex, however, the planes in the case of Ni-complex exported less electronic localization around N1 and N2 donor sites of TSC.

#### Reduced density gradient approximation

**Fig. 10:**
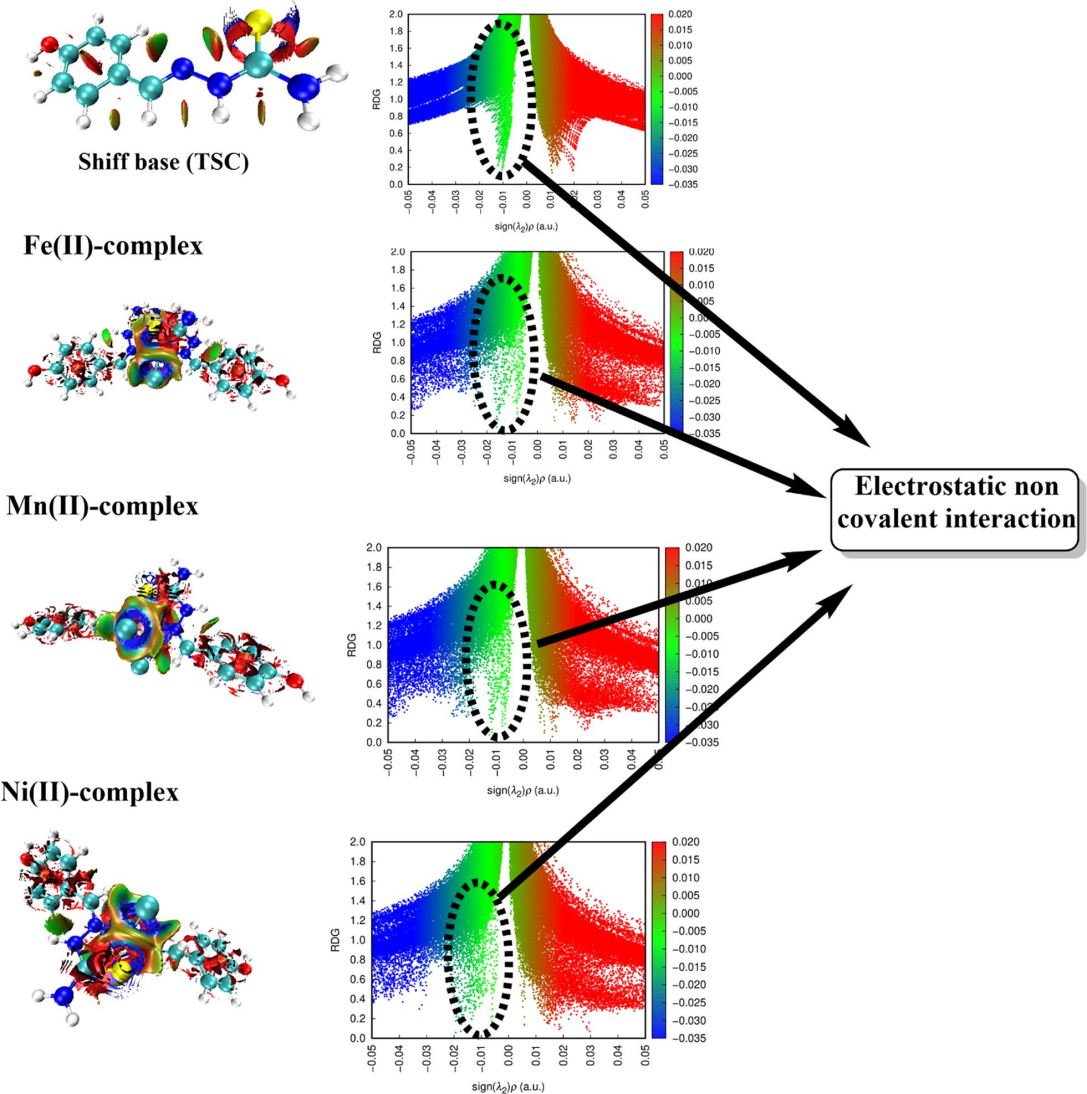
RDG and NCI map for studied TSC and its metal complexes (on the left side colored molecular interaction types were presented, and the RDG plot of colored spikes appeared on the right side), blue spikes refer to H-bond formation (blue spikes disappear in the chart), green spikes refer to electrostatic interactions and red spikes refer to repulsion interaction

The QTAIM study is unable to identify any of the expected weak non-covalent interactions, especially intramolecular hydrogen bonds [70, 71]. After evaluating the isosurfaces of the reduced density gradient (r), plots were made for the studied metal complexes. Figure 10 shows a complete map to discuss the ability of molecular linkage with some favorable non-covalent interactions. The generated plots show the reduced density gradient (RDG) versus the electron density multiplied by the sign of the second Hessian and gradient isosurfaces, illustrating intramolecular interactions for the current complexes. The observed results indicate the presence of some variety in the RDG plot for each complex. This is attributed to the geometrical structure of the M-L complex and is mainly a consequence of coordination environment of the metal involved based on its bonding strength. The presence of the L-aromatic rings in the structure may export some information about the spike distribution related to each interaction type. For some details, the color scale of RDG plot insight an interaction type, whereas the electronic density integrated parameter (sign λ_2_)ρ reaches to value < 0. The favorable interactions (H-bond in blue spike and vdW in green spike) are mostly predominant. On the other hand, changing the (sign λ_2_)ρ parameter to be > 0 leads to the generation of unfavorable (steric with red spike) interaction. Higher steric hindrance appears in the region of (sign λ_2_)ρ < 0.03 in all proposed metal complexes, resulting from the restriction exported in the membered phenyl ring and also some crowd in the M-L coordination area. However, this unfavorable interaction can be compensated with the electrostatic attraction forces (vdW).

### Protein-binding affinity

**Fig. 11:**
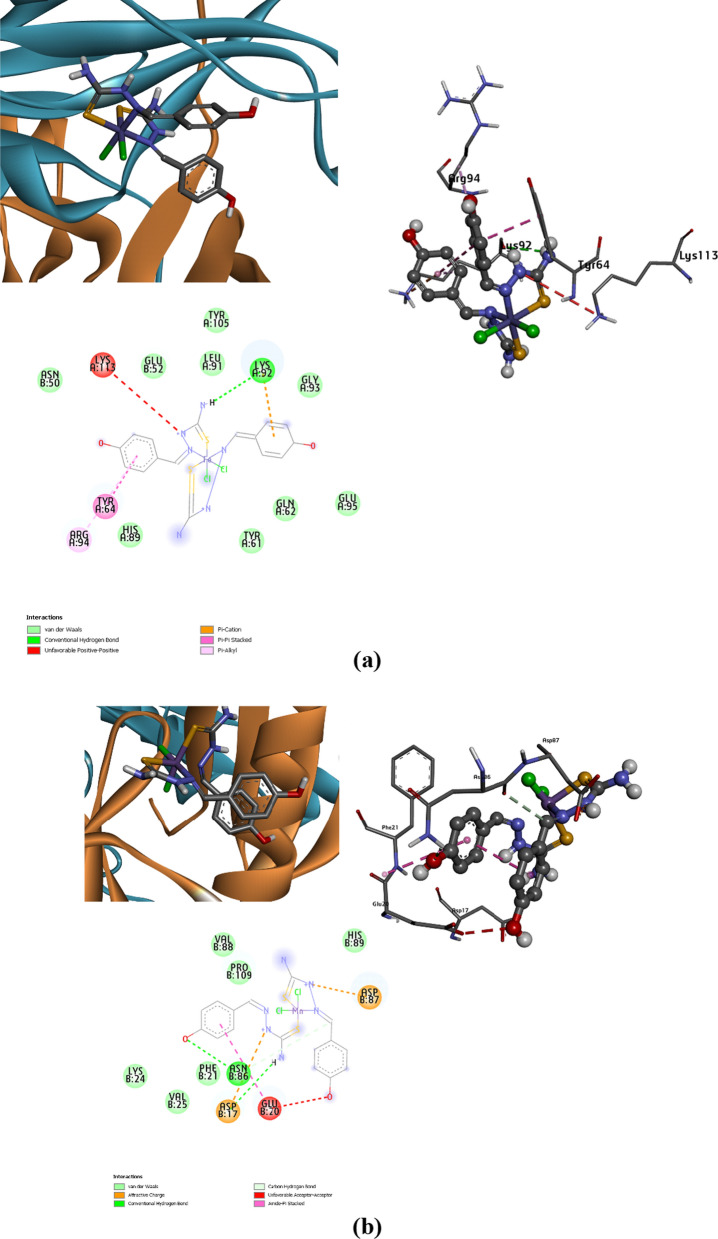

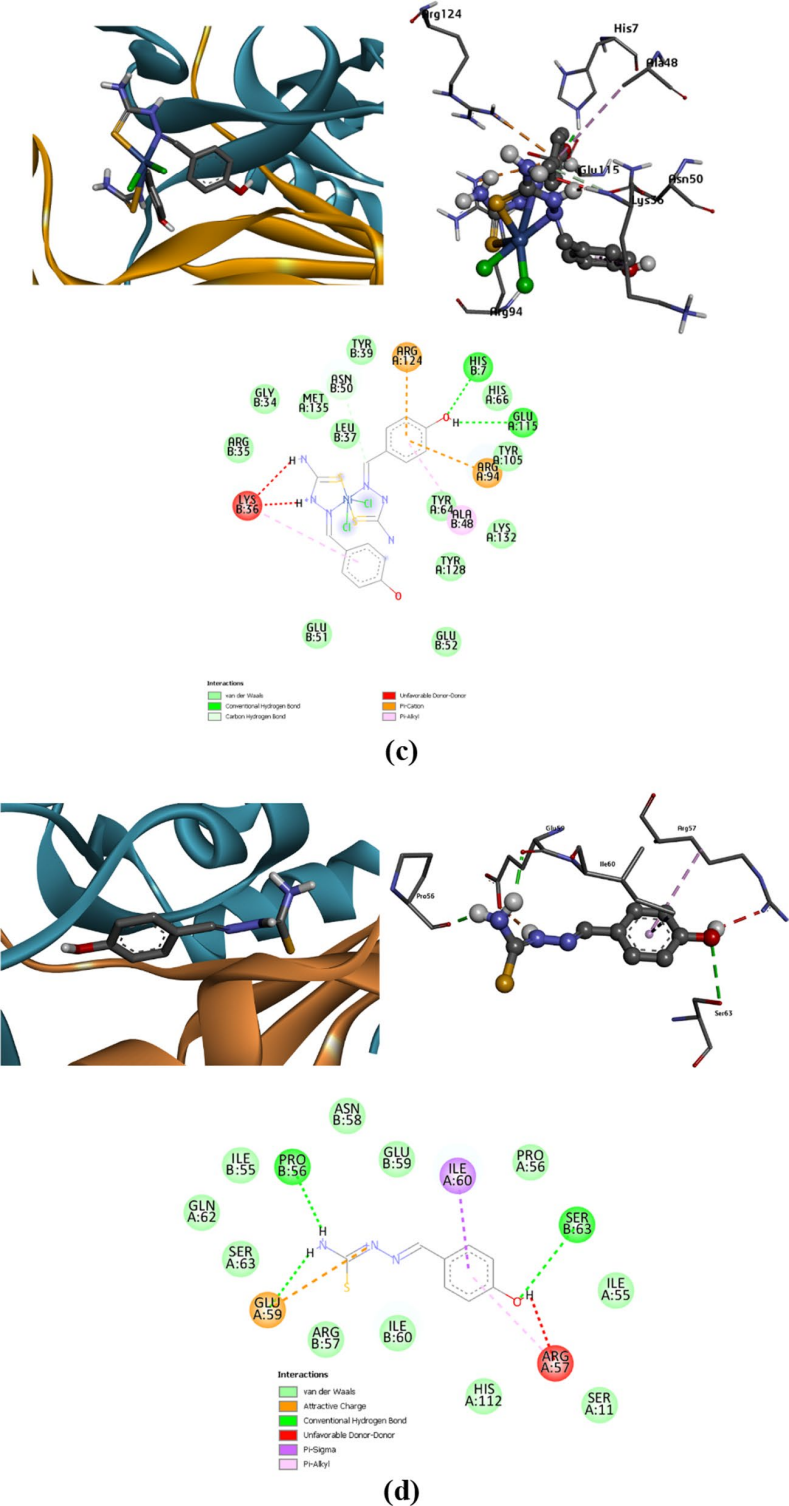
Docking score and 3D, 2D interactions of the the synthesized complexes with target bacterial enzyme 4JH9, **a** Fe(II), **b** Mn(II), **c** Ni(II), complexes, and **d** schiff base TSC

**Fig. 12:**
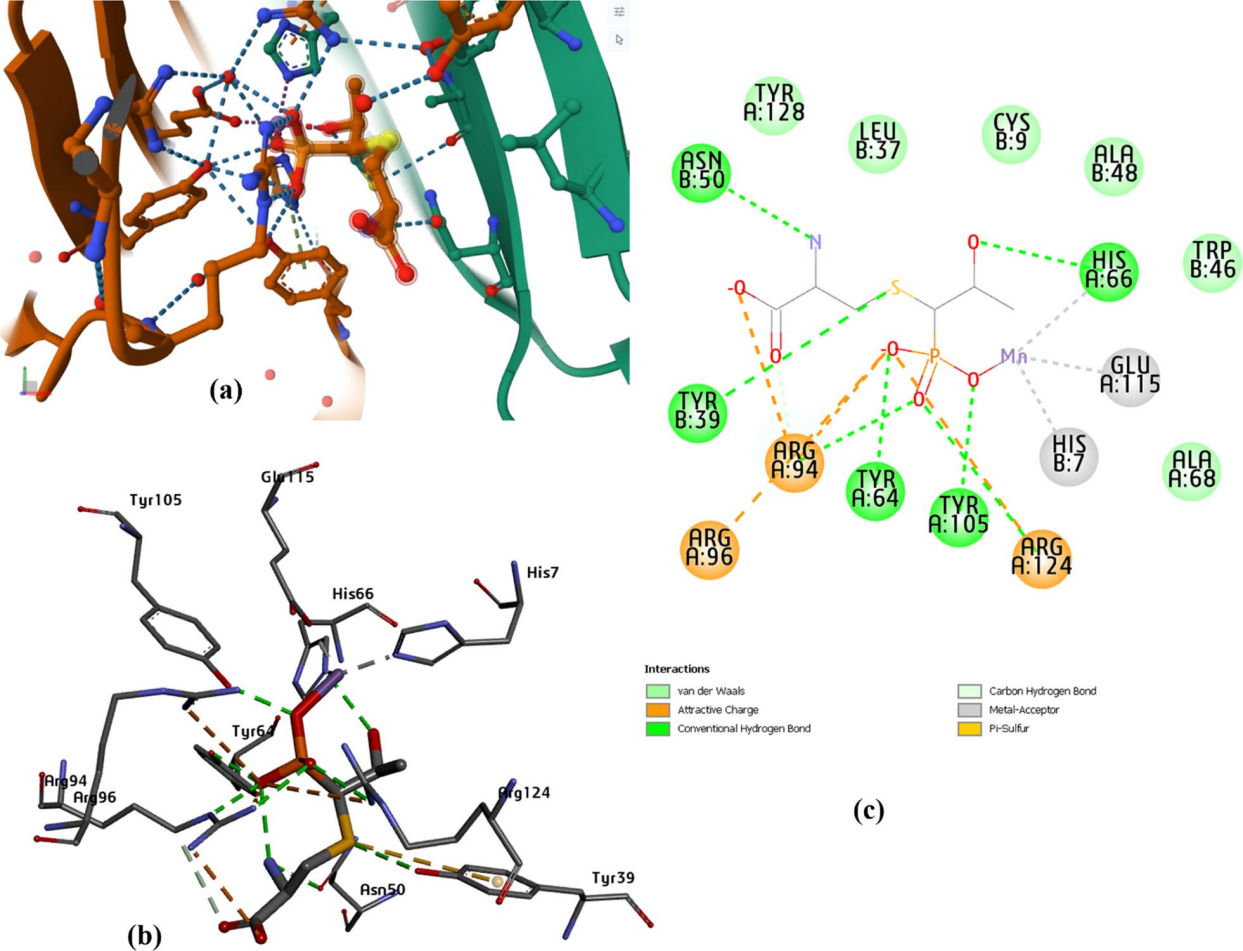
Docking Results of the co-crystalized control, **a** crystallographic protein structure including the control, **b** 3D-binding mode of the control in the active site, **c** 2D-map representing the type of interactions present through control-docking

The docking approach primarily follows a vital route for incredibly efficient outcomes [72–74]. Possible Interaction between the synthesized complexes and the microbial transferase enzyme 4JH9 was described with the docking simulation study with the lowest energy generation to provide the most accurate description of the binding mechanism of the complexed macromolecular structure [75, 76]. Protein active site detection, which was illustrated to get the site-best score, is necessary for proper binding. Figure 11 illustrates the binding affinity of the proposed metal complexes with the target protein, where the obtained docking results generated the lowest binding energy exhibited with binding of [Ni(L)_2_Cl_2_] (− 7.1 kcal/mol), then [Fe(L)_2_Cl_2_], and [Mn(L)_2_Cl_2_] complexes with values − 6.4, and − 6.3 kcal/mol, respectively. The best-predicted docking results were exported from several favorable interactions that appeared during docking analysis. Such the conventional H-bond and carbon hydrogen bond, which are strong non-covalent bonds, appears in Ni(II) complex within HIS.B 7, GLU.A 115 and ASN.B 50. In Fe(II) complex, the significant H-bond interactions present within LYS.A 92. This type of interaction observed within ASN.B 86 and ASP.B 17 in Mn(II) complex. Other interaction types such as vdW, attractive charges, π–π stacked, π-alkyl and π–π T-shaped linked with all the parts of the metal complexes. Some unfavorable interactions are exhibited in some active sites such as positive-positive, acceptor-acceptor and donor-donor interactions which may destabilize the docked complexes. These types of unfavorable Bumps may decrease the pose stability in its active site, while other attraction forces can compensate for this instability conformation. For precise affinity prediction, the binding affinity of the studied Schiff base ligand TSC was evaluated as shown in Fig. 11d. The binding affinity in the predicted active site is about − 5.7, where the H-bonding interacting amino acids involve PRO.B 56, GLU.A 59 and SER.B 63. The docking findings summarized the binding affinity, that occurs for these studied conformers, which is mostly close in behavior leading to the prediction of the potency inhibition effect [77]. Figure 12 represents the co-crystalized ligand within the macromolecular target protein, where its docking results give some similarity to the tested complexes. To compare the in silico docking results for [NiL_2_Cl_2_] complex, it was found the common amino acids in the active site referred to HIS.A 7, GLU.A 45, TYR. A 64, ALA.A 84, ARG.A 94, ARG.A 124 and ASN.B 50. In case of [FeL_2_Cl_2_], the common interacted amino acids presented in TYR.A 64 and ARG.A 94. These findings summarize the inhibition prediction of the most interactive [NiL_2_Cl_2_], then [FeL_2_Cl_2_] complexes as confirmed from the binding energy values.

### Evaluation of MIC analysis

The study using turbidity analyses to assess the impact of FeL_2_Cl_2_, NiL_2_Cl_2_, and MnL_2_Cl_2_ on the growth of *B. cereus* and *K.pneumoniae* isolates in liquid culture media. The current study found that MnL_2_Cl_2_ have higher MICs of *B. cereus* and *K.pneumoniae* compared to FeL_2_Cl_2_, and NiL_2_Cl_2_, with MICs of ≤ 128–256 µgmL–1, and ≤ 64–128 µgmL–1, respectively. While MnL_2_Cl_2_ had lower MICs (≤ 16–32 µgmL–1) against *B. cereus* and *K.* *pneumoniae*. MIC data were evaluated and tabulated in Table 5**.**

**Table 5:** MIC of FeL_2_Cl_2_, NiL_2_Cl_2_, and MnL_2_Cl_2_

Isolations	Sub-MIC (μgmL^−1^)		
	**FeL**_**2**_**Cl**_**2**_	**NiL**_**2**_**Cl**_**2**_	**MnL**_**2**_**Cl**_**2**_
*B. cereus*	≤ 128	≤ 64–128	≤ 16–32
*K. pneumoniae*	≤ 256	≤ 128	≤ 32
*B. cereus*	≤ 128–256	≤ 64	≤ 32
*K. pneumoniae*	≤ 128–256	≤ 64	≤ 16–32

### Antibacterial activity of [FeL_2_Cl_2_], [NiL_2_Cl_2_], and [MnL_2_Cl_2_]

**Fig. 13:**
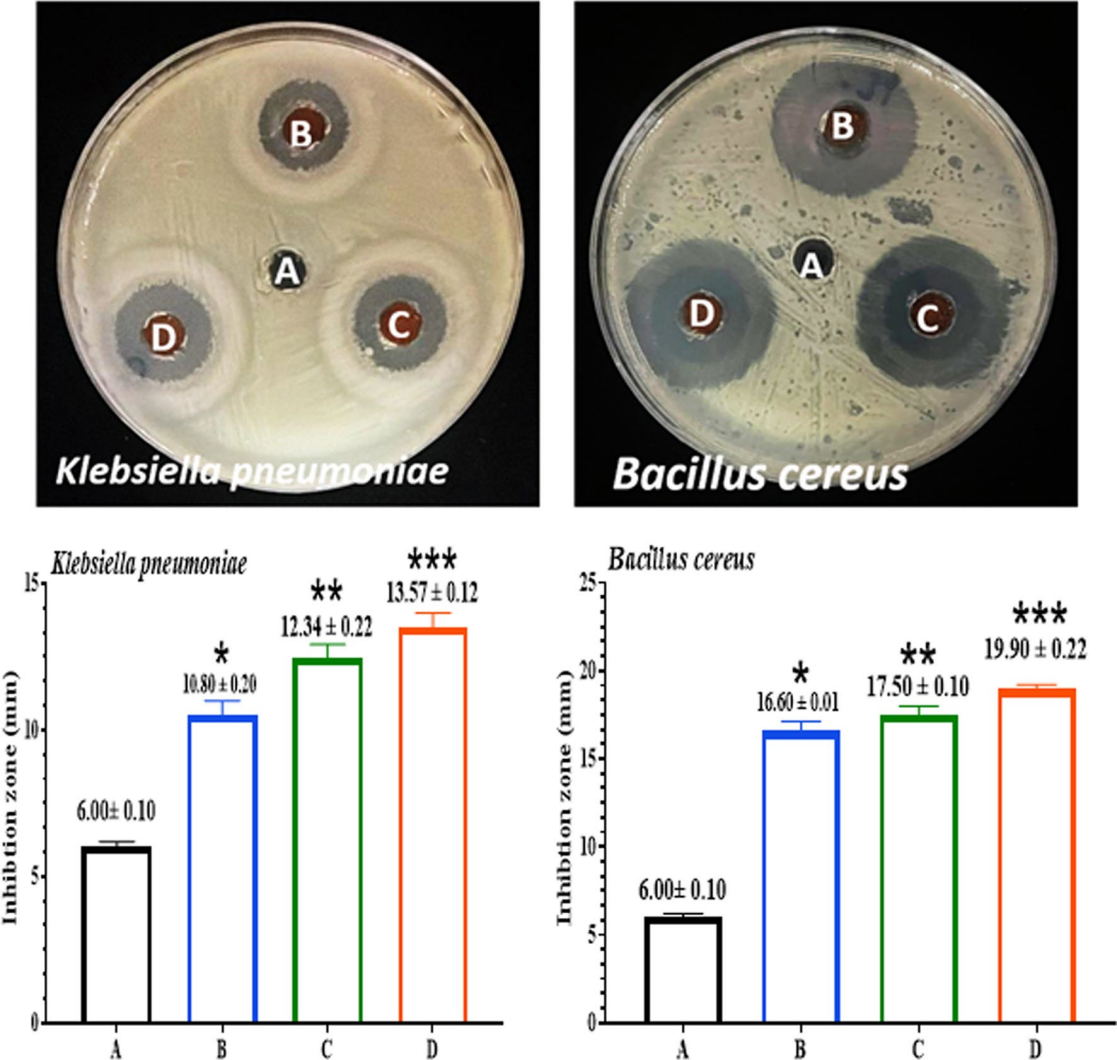
Antibacterial activity of [FeL_2_Cl_2_] against *B. cereus& K.pneumoniae.* **A** (DW as a negative control). **B** (25µgmL^−1^). **C** (50µgmL^−1^). **D** (100µgmL^−1^). The results were represented as mean ± SD of three independent experiment. The asterisks indicated that there was a noteworthy variation from the negative control. *p ≤ 0.05, **p ≤ 0.01, ***p ≤ 0.001

**Fig. 14:**
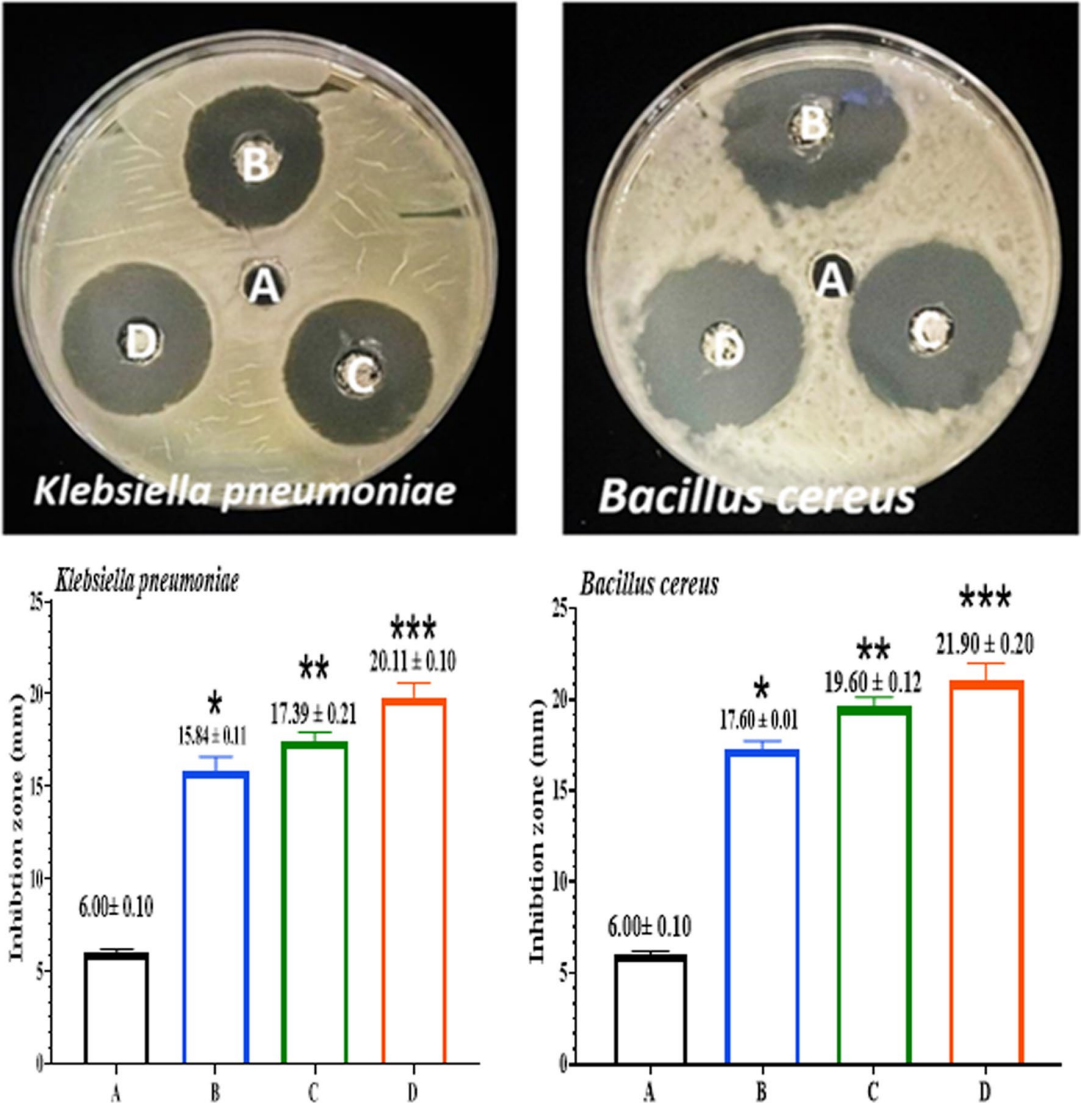
Antibacterial activity of [NiL_2_Cl_2_] against *B. cereus* and* K.pneumoniae*. **A** (DW as a negative control). **B** (25µgmL^−1^). **C** (50µgmL^−1^). **D** (100µgmL^−1^). The results were represented as mean ± SD of three independent experiment. The asterisks indicated that there was a noteworthy variation from the negative control. *p ≤ 0.05, **p ≤ 0.01, ***p ≤ 0.001

**Fig. 15:**
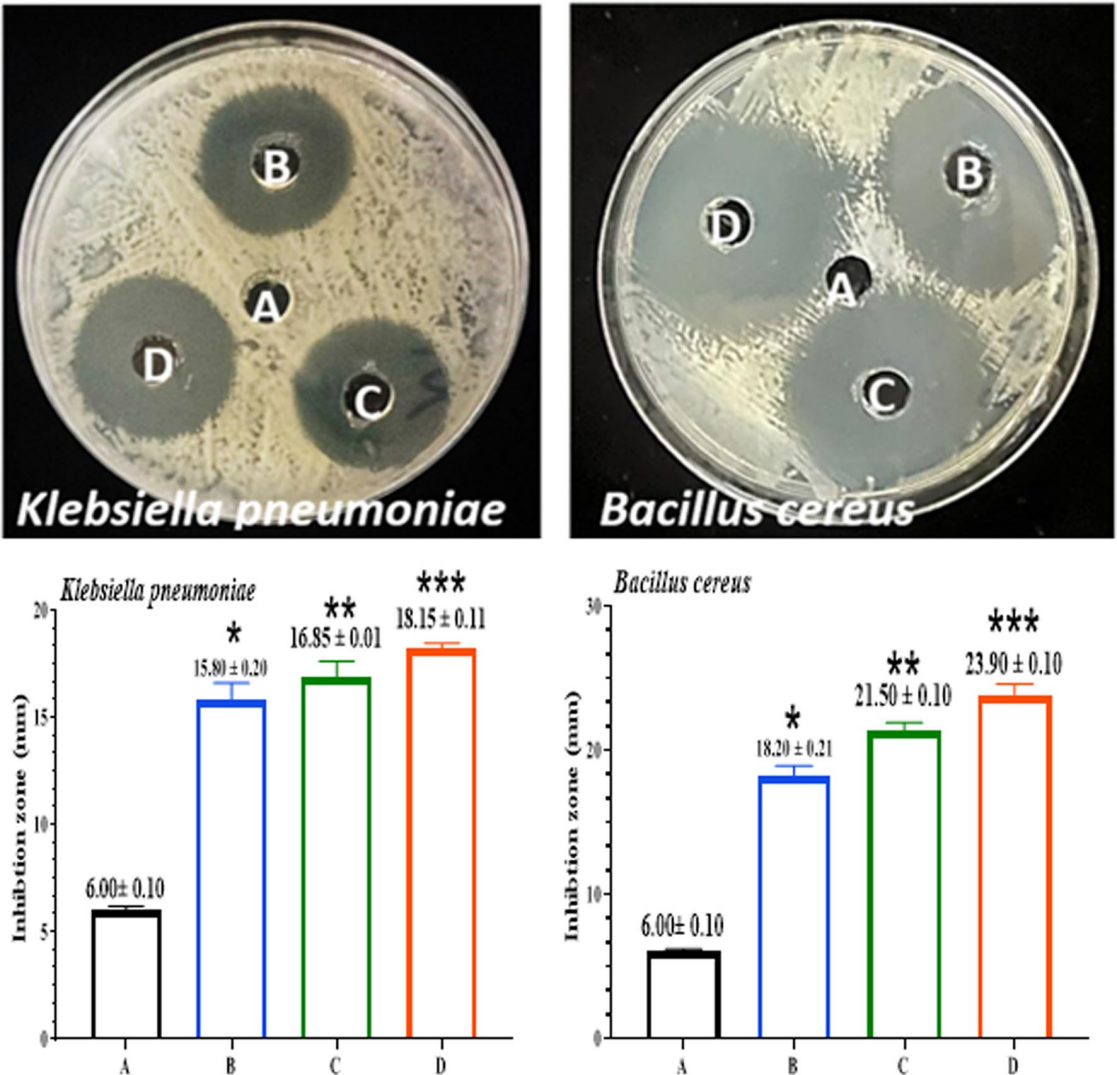
Antibacterial activity of [MnL_2_Cl_2_] against *B. cereus* and* K.* *pneumoniae.* **A** (DW as a negative control). **B** (25µgmL^−1^). **C** (50µgmL^−1^). **D** (100µgmL^−1^). The results were represented as mean ± SD of three independent experiments. The asterisks indicated that there was a noteworthy variation from the negative control. *p ≤ 0.05, **p ≤ 0.01, ***p ≤ 0.001

The evolution-inhibitory effects of the Compounds [FeL_2_Cl_2_], [NiL_2_Cl_2_], and [MnL_2_Cl_2_] against *B. cereus *and* K. pneumoniae* were indicated by the antibacterial properties of the compounds at varied concentrations (25,50,100 µg/ml). According to Figs. 13, 14, 15. Gram-positive bacteria are more susceptible to the compounds than Gram-negative bacteria. The outstanding antibacterial activity of compounds against all tested isolates in this context is shown in Figs. 13–15. The observations of [FeL_2_Cl_2_], [NiL_2_Cl_2_], and [MnL_2_Cl_2_] corroborate the material's effectiveness in treating bacterial infections. [FeL_2_Cl_2_], [NiL_2_Cl_2_], and [MnL_2_Cl_2_] were proven to be biocompatible, making them suitable for integration into a variety of industrial and biological applications [71]. Due to the presence of hydrophilic glycoproteins and the physical contact of compounds [FeL_2_Cl_2_], [NiL_2_Cl_2_], and [MnL_2_Cl_2_] with the cell walls of gram-positive bacteria, the cell walls were damaged, allowing compounds to enter gram-positive bacteria. Additionally, the heightened membrane permeability in gram-positive bacteria causes compounds to leak, which facilitates the entry of compounds [78]. Compounds caused lipid peroxidation and the production of reactive oxygen species, which killed bacterial cells.

### Prediction of binding affinity of the gram-positive *S. aureus* compared with experimental the gram positive of *B. cereus*

This study was added to estimate the inhibition effect of the designed metal complexes on the other type of microorganism (*S. aureus*) that is referred to gram positive bacteria type (thick wall type). It was found that the binding affinity of the complexes with Gram-positive bacterial thymidylate kinase TMK (ID: 4XWA) take the order MnL_2_Cl_2_ > NiL_2_Cl_2_ > FeL_2_Cl_2_ where the binding energy values were considered as − 9.4 kcal/mol for MnL_2_Cl_2_, − 9.0 kcal/mol for NiL_2_Cl_2_ and − 8.6 kcal/mol for FeL_2_Cl_2_ (the lower binding energy values give higher binding affinity in the active site). Supplementary Figs. 5–7 display the molecular docking analysis of the metal complexes against the selected type of gram-positive bacteria. Supplementary Fig. 8 represents the control inhibitor within its active site in the bacterial target protein Antibacterial experiments measured the inhibition efficiency of the metal complexes with two types of bacterial strains (gram positive and gram negative), it was found that gram positive bacteria (*B. cereus*) give the same results with the computational analysis. The docking results were performed for *S. aureus* (ID: 4XWA) as the same methodology of docking with *B. cereus* (ID: 4JH9).

### Bacterial biofilm inhibition

**Fig. 16:**
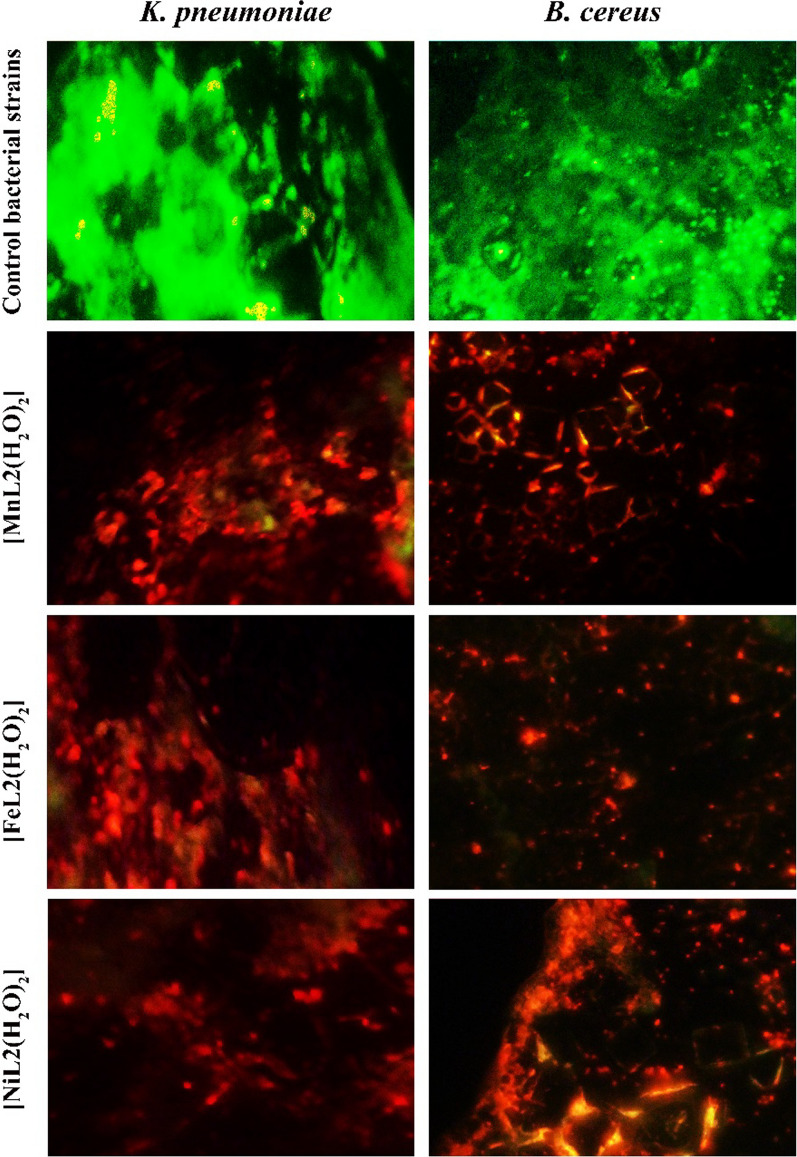
[FeL_2_Cl_2_], [NiL_2_Cl_2_], and [MnL_2_Cl_2_] reduces the level of bacterial biofilm formation

To evaluate the effect of [FeL_2_Cl_2_], [NiL_2_Cl_2_], and [MnL_2_Cl_2_] on bacterial biofilm inhibition, fluorescent microscopy was used. The results of the current study demonstrated the inhibition of bacterial antibiofilm formation of [FeL_2_Cl_2_], [NiL_2_Cl_2_], and [MnL_2_Cl_2_] at a concentration of 100 μg/mL. As indicated in Fig. 16, the Syto 9 green fluorescence dye stained healthy bacteria, whereas propidium iodide red fluorescence dye stained the dead bacterial strains suffering from damaged membranes. The results showed the control of untreated bacteria strains were aggregated and covered with a mature biofilm structure, while the bacterial strains that were treated with [FeL_2_Cl_2_], [NiL_2_Cl_2_], and [MnL_2_Cl_2_] MWCNTs were less aggregated and had a less dense biofilm covering. Taken together, the results of the current study reveal the potential biofilm formation inhibition of [FeL_2_Cl_2_], [NiL_2_Cl_2_], and [MnL_2_Cl_2_].

### Anti-oxidant activity of [FeL_2_Cl_2_], [NiL_2_Cl_2_], and [MnL_2_Cl_2_]

**Fig. 17:**
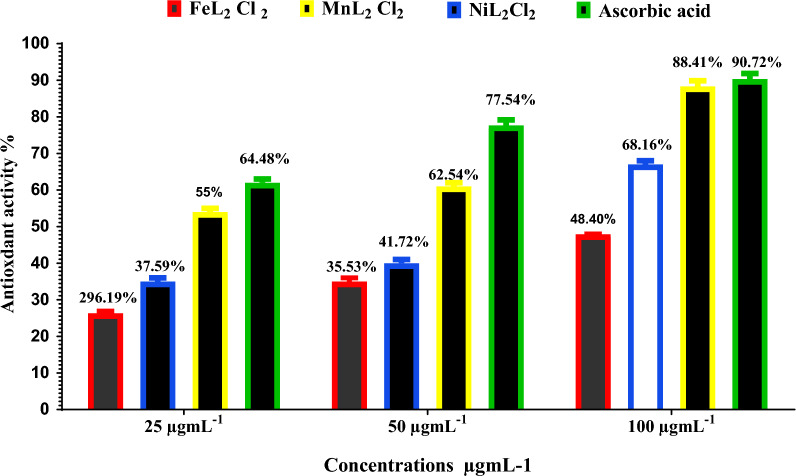
Antioxidant activity of [FeL_2_Cl_2_], [NiL_2_Cl_2_], and [MnL_2_Cl_2_]by DPPH assay

Compounds [FeL_2_Cl_2_], [NiL_2_Cl_2_], and [MnL_2_Cl_2_] that have higher antioxidant activity than the control exhibits free radical scavenging characteristics, indicating their ability to interact and neutralize free radicals to prevent them from causing damage (Fig. 17). The IC₅₀ values were 38.33 μg/ml for FeL₂Cl₂, 27.35 μg/ml for NiL₂Cl₂, and 34.79 μg/ml for MnL₂Cl₂. The IC₅₀ for Ascorbic acid is 22.35 μg/ml. These compounds also act as scavengers of the DPPH + radical due to a decrease in these radicals. To evaluate a substance’s efficacy and investigate its antioxidant qualities, a method has been found and established that is both comprehensive and sufficient. The numerous mechanisms and activities of distinct antioxidants can be taken into consideration when assessing the antioxidant strength utilizing a variety of measurement methodologies [43]. The current study employed DPPH techniques to evaluate a compound's ability to remove free radicals. The DPPH assay has been widely utilized using a range of extract concentrations to evaluate the compound's antioxidant effectiveness because it uses relatively common equipment. It also yields results quickly and consistently. The most efficient, straightforward, and accurate DPPH techniques have been identified through a comparison of previously published methodologies for evaluating antioxidant capacity.

We summarized the potential effect of the current metal complexes based on their bioactivity in the medicinal approach and displayed the results in Table 6.

**Table 6:** the docking core and biological data of the studied metal complexes

Complex	B.E^a^ (kcal/mol) *B. cereus S. aureus* (4JH9) (4XWA)		Antibacterial activity (25µgmL^−1^) *B.cereus K.pneumoniae*		Anti-oxidant activity IC₅₀ (μg/ml)
[FeL_2_Cl_2_]	− 7.1	− 9.4	16.60 ± 0.01	10.80 ± 0.20	38.33
[NiL_2_Cl_2_]	− 6.4	− 9.0	17.60 ± 0.01	15.84 ± 0.11	27.35
[MnL_2_Cl_2_]	− 6.3	− 8.6	18.20 ± 0.20	15.80 ± 0.21	34.79

^a^Binding energy of the two target bacterial protein were selected based on the type of the bacterial wall (thick wall gram-positive bacteria)

## Conclusion

In this paper new Schiff Base ligand (L) complexes with the general formula [M(L)_2_Cl_2_] where M = Ni(II), Mn(II), and Fe(II), were synthesized. The elemental analysis, magnetic susceptibility, molar conductivity, FTIR, and UV–visible electronic spectral observations and the configurations were performed to coordinate the Schiff base through the nitrogen and sulfur atoms. From the obtained results, it can be concluded that the bonding of all metal ions (M (II)) to the ligand (L) leads to form an octahedral structure. DFT calculations exhibited a significant behavior towards reactivity and the favorable electronic transitions for Ni(II) complex based on the energy gap (EGAP equal 1.190 eV). Also, other quantum chemical parameters were calculated to predict the complex’s stability. The molecular docking approach estimated a potent inhibition effect of the studied complexes towards the target bacterial enzyme related to the co-crystallized reference. In silico study of the designed complexes with *Bacillus cereus* evaluated the potent inhibition activity of the metal complexes, especially for [NiL_2_Cl_2_] with higher binding affinity (− 7.1 kacl/mol). Further in silico study was performed on other bacterial strain of gram-positive type (*s. aureus*), the finding supported the experimental results with higher bacterial inhibition in presence of MnL_2_Cl_2_ complex. The docking results evolved a more stable Mn(II) conformer (− 9.4 kcal/mol) based on the binding affinity in the estimated active site. comparing these results with experimental ones, MnL_2_Cl_2_ had lower MICs (≤ 16–32 µgmL^–1^) against *B. cereus& Pneumonia* in MIC analysis. Using fluorescent microscopy study revealed the potential biofilm formation inhibition of [FeL_2_Cl_2_], [NiL_2_Cl_2_], and [MnL_2_Cl_2_]. Anti-oxidant activity evaluated a significant result in complex behavior to remove free radicals. The IC₅₀ values of the antioxidant activity were 38.33 μg/ml for FeL₂Cl₂, 27.35 μg/ml for NiL₂Cl₂, and 34.79 μg/ml for MnL₂Cl₂, where the IC₅₀ for Ascorbic acid is 22.35 μg/ml. The biological studies in this research may applied on other series of metal candidates to have a future direction as potent inhibitors in medicinal strategy.

## Supplementary Information

Additional file 1.

## Data Availability

The datasets generated and/or analyzed during the current study are available in the supplementary file.

## Abbreviations

TSC: Thiosemicarbazone
FTIR: Fourier transform Infra-red
MEP: Molecular electrostatic behavior
DPPH: 2,2-Diphenyl-1-picryl-hydrazyl-hydrate
DFT: Density functional theory
FMOs: Frontier molecular orbitals
HOMO: Highest occupied molecular orbital
LUMO: Lowest unoccupied molecular orbital
GP: Gram-positive
GN: Gram-negative
MIC: Minimum inhibitory concentration
SD: Standard deviation
ELF: Electron localization function
QTAIM: Quantum atoms in molecule
RDG: Reduced density gradient
