# Supplementary material for: Structural and topological analysis of thiosemicarbazone-based metal complexes: computational and experimental study of bacterial biofilm inhibition and antioxidant activity
Source: BMC Chem. 2025 Jan 24;19(1):24. doi: 10.1186/s13065-024-01338-5 (PMC11762858; doi:10.1186/s13065-024-01338-5)
Supplement: Supplementary file 1 — Additional file 1. [file 13065_2024_1338_MOESM1_ESM.docx]

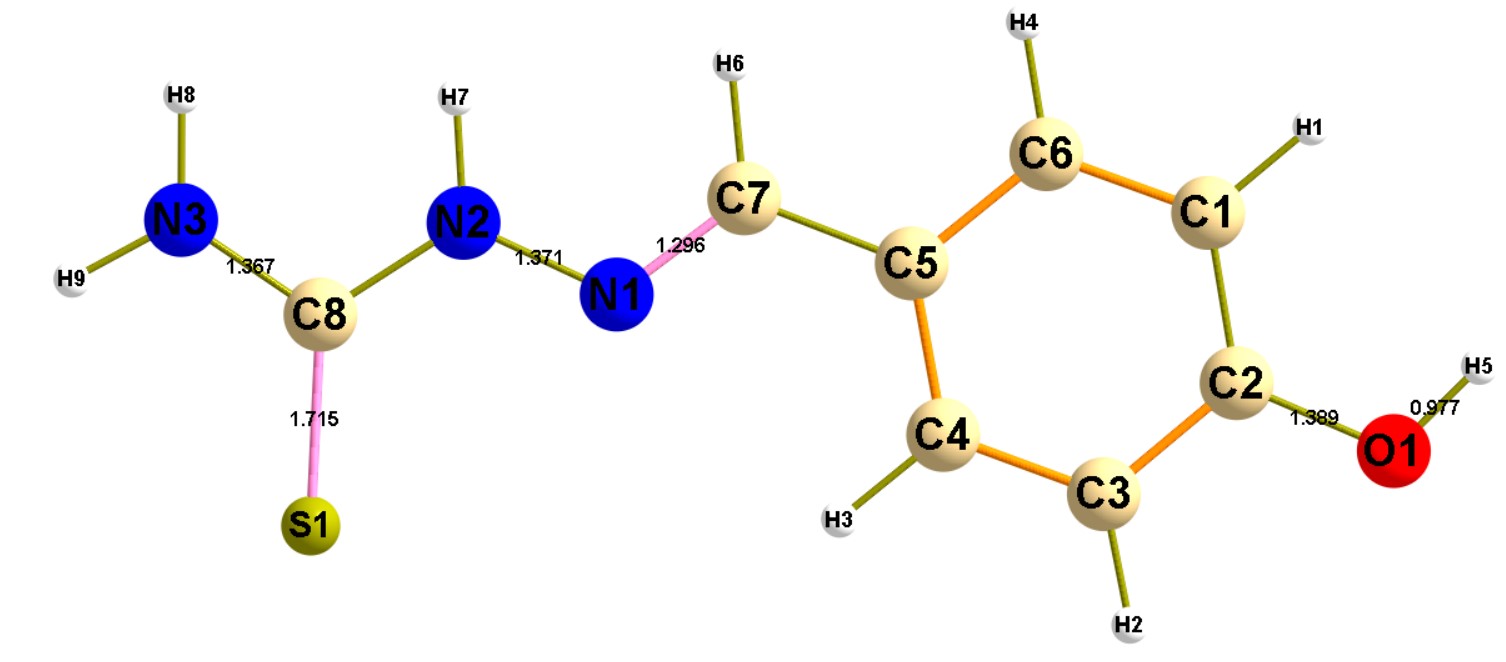


**Supplementary figure 1. Optimized structure of TSC Schiff base with intersetd bond lengths**


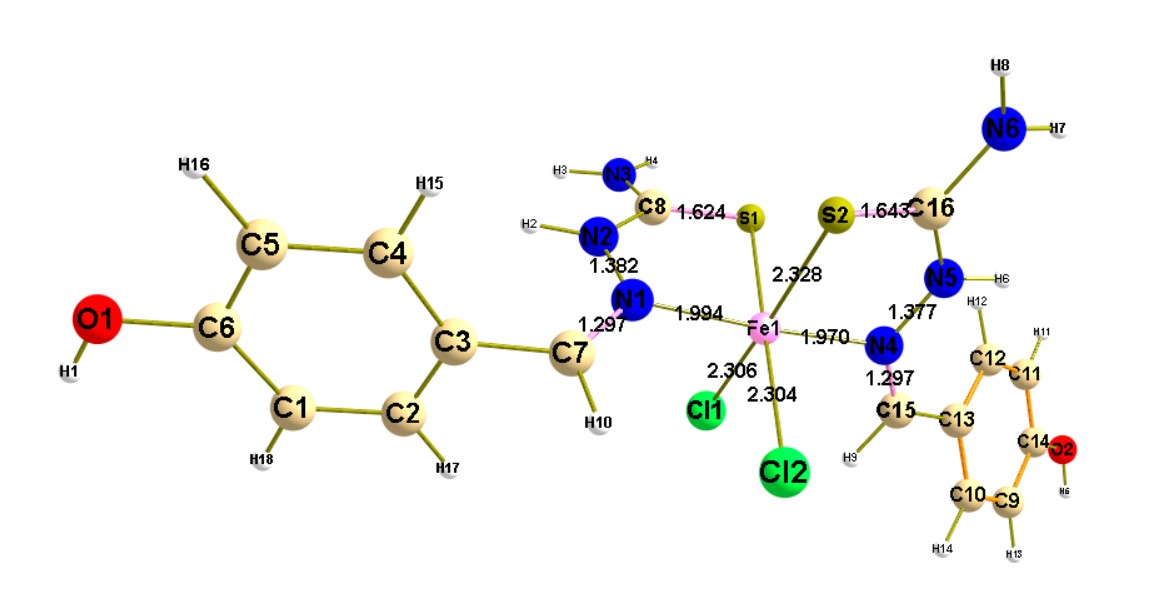


**Supplementary figure 2. Optimized structure of Fe (II) complex with intersetd bond lengths**


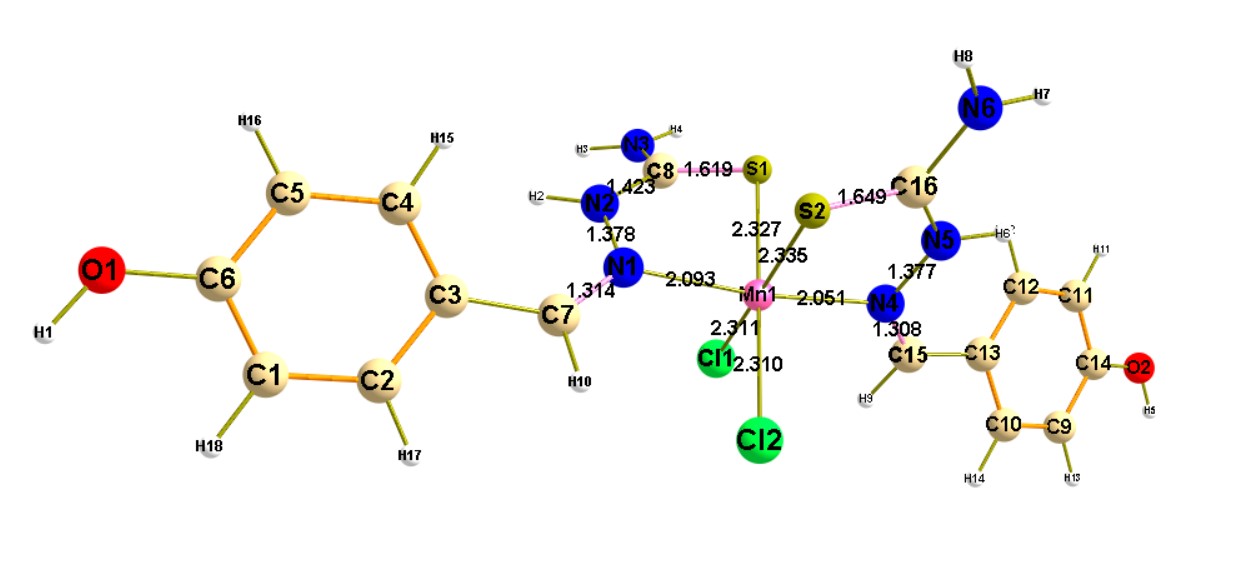
 **Supplementary figure 3. Optimized structure of Mn (II) complex with intersetd bond lengths**


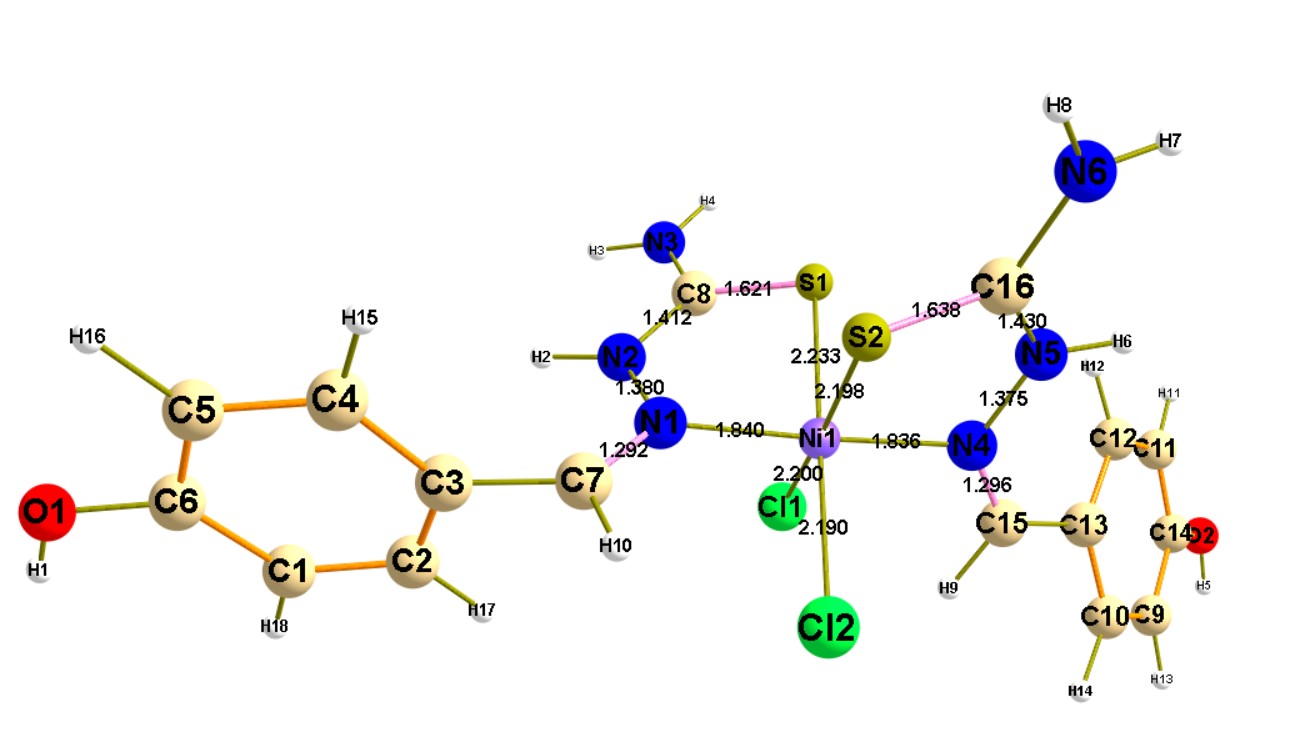


**Supplementary figure 4. Optimized structure of Ni (II) complex with intersetd bond lengths**

**Supplementary table 1. Cartesian coordinates of Schiff base TSC**

| **Atom** | **x** | **y** | **z** |
| --- | --- | --- | --- |
|  |  |  |  |
| C | -3.6482 | -1.01014 | -6.2E-05 |
| C | -3.88806 | 0.369057 | 5.3E-06 |
| C | -2.81734 | 1.276919 | 7.57E-05 |
| C | -1.51154 | 0.805023 | 7.7E-05 |
| C | -1.24732 | -0.58392 | -9.5E-07 |
| C | -2.33281 | -1.47794 | -7.2E-05 |
| O | -5.16647 | 0.911409 | 5.41E-05 |
| C | 0.114924 | -1.10331 | -9.4E-06 |
| N | 1.144197 | -0.31621 | 1.89E-05 |
| N | 2.389943 | -0.88944 | 2.88E-06 |
| C | 3.544902 | -0.13708 | 1.43E-05 |
| S | 3.612766 | 1.576869 | -9E-05 |
| N | 4.680575 | -0.89735 | 0.000111 |
| H | -4.47726 | -1.71272 | -9.6E-05 |
| H | -3.03518 | 2.33811 | 0.000127 |
| H | -0.67492 | 1.494109 | 0.000127 |
| H | -2.14806 | -2.54867 | -0.00013 |
| H | -5.85648 | 0.220418 | -0.0003 |
| H | 0.228261 | -2.19617 | -3.8E-05 |
| H | 2.456196 | -1.90769 | 1.15E-05 |
| H | 4.68244 | -1.90763 | 0.000178 |
| H | 5.562203 | -0.4116 | 3.53E-05 |

**Supplementary table 2. Cartesian coordinates of Fe(II) complex**

| **Atom** | **x** | **y** | **z** |
| --- | --- | --- | --- |
|  |  |  |  |
| C | 5.9214 | -2.1353 | -0.0654 |
| C | 4.5964 | -1.8333 | -0.3681 |
| C | 4.2112 | -0.5118 | -0.6109 |
| C | 5.1635 | 0.5173 | -0.5637 |
| C | 6.5011 | 0.2096 | -0.2803 |
| C | 6.8818 | -1.1157 | -0.0243 |
| O | 8.1676 | -1.4001 | 0.2619 |
| H | 8.46 | -2.3037 | 0.451 |
| C | 2.7901 | -0.2677 | -0.8961 |
| N | 1.9676 | 0.2399 | -0.0314 |
| N | 2.3808 | 0.5915 | 1.2402 |
| C | 1.4193 | 0.9236 | 2.2336 |
| N | 1.7812 | 0.934 | 3.6045 |
| H | 3.3552 | 0.4548 | 1.5453 |
| H | 2.7324 | 0.6747 | 3.8993 |
| H | 1.0779 | 1.1472 | 4.3285 |
| S | -0.1227 | 1.2418 | 1.8342 |
| C | -6.057 | -2.3096 | -0.3714 |
| C | -4.7401 | -1.9979 | -0.709 |
| C | -6.2255 | -0.2401 | 0.8683 |
| C | -4.9079 | 0.0783 | 0.5267 |
| C | -4.1476 | -0.8008 | -0.2697 |
| C | -6.801 | -1.4359 | 0.43 |
| O | -8.0552 | -1.7498 | 0.8103 |
| H | -8.4661 | -2.5935 | 0.5732 |
| C | -2.6901 | -0.6394 | -0.4974 |
| N | -1.9652 | 0.4361 | -0.4676 |
| N | -2.4405 | 1.7276 | -0.5032 |
| C | -1.5179 | 2.7775 | -0.7977 |
| N | -1.978 | 4.1121 | -0.9291 |
| H | -3.4415 | 1.9483 | -0.5437 |
| H | -2.9669 | 4.3452 | -0.7649 |
| H | -1.3282 | 4.8651 | -1.202 |
| S | 0.0522 | 2.4458 | -1.1522 |
| Fe | -0.0089 | 0.284 | -0.2902 |
| Cl | -0.0342 | -1.8194 | 0.6535 |
| Cl | 0.03 | -0.7264 | -2.361 |
| H | -2.184 | -1.5845 | -0.5961 |
| H | 2.4447 | -0.6554 | -1.8342 |
| H | -6.786 | 0.4265 | 1.5114 |
| H | -4.4893 | 0.9702 | 0.9635 |
| H | -6.4848 | -3.2455 | -0.7079 |
| H | -4.17 | -2.7104 | -1.2943 |
| H | 4.8776 | 1.5441 | -0.7582 |
| H | 7.2416 | 0.9991 | -0.2558 |
| H | 3.8592 | -2.6275 | -0.3907 |
| H | 6.1957 | -3.1623 | 0.1379 |

**Supplementary table 3. Cartesian coordinates of Mn(II) complex**

| Atom | x | y | z |
| --- | --- | --- | --- |
|  |  |  |  |
| C | 6.638746 | -1.65514 | -0.68901 |
| C | 5.248788 | -1.5504 | -0.84027 |
| C | 4.515409 | -0.45691 | -0.33132 |
| C | 5.267584 | 0.56176 | 0.329761 |
| C | 6.675917 | 0.461492 | 0.458088 |
| C | 7.360998 | -0.64962 | -0.04661 |
| O | 8.705528 | -0.74148 | 0.081061 |
| H | 9.210737 | -1.49595 | -0.26298 |
| C | 2.998546 | -0.46356 | -0.59412 |
| N | 2.041211 | 0.066858 | 0.132772 |
| N | 2.302412 | 0.593285 | 1.379691 |
| C | 1.223022 | 1.033791 | 2.196502 |
| N | 1.34248 | 1.026864 | 3.604549 |
| H | 3.20551 | 0.426904 | 1.83989 |
| H | 2.201164 | 0.685124 | 4.053795 |
| H | 0.533688 | 1.283284 | 4.191495 |
| S | -0.19581 | 1.477609 | 1.555284 |
| C | -6.50012 | -1.9835 | -0.15109 |
| C | -5.12283 | -1.88056 | -0.37355 |
| C | -6.53426 | 0.273377 | 0.69706 |
| C | -5.14528 | 0.388878 | 0.457954 |
| C | -4.40911 | -0.70061 | -0.089 |
| C | -7.21209 | -0.91327 | 0.3924 |
| O | -8.53631 | -1.02721 | 0.6402 |
| H | -9.02625 | -1.84521 | 0.459397 |
| C | -2.89613 | -0.75834 | -0.24356 |
| N | -2.04464 | 0.203465 | -0.48757 |
| N | -2.41639 | 1.474742 | -0.86449 |
| C | -1.42307 | 2.373026 | -1.37341 |
| N | -1.79373 | 3.687622 | -1.7498 |
| H | -3.40178 | 1.759164 | -0.92425 |
| H | -2.76396 | 4.010768 | -1.63364 |
| H | -1.09317 | 4.339653 | -2.13345 |
| S | 0.13374 | 1.893554 | -1.62992 |
| Mn | -0.01666 | 0.017342 | -0.24737 |
| Cl | -0.15628 | -1.82186 | 1.14465 |
| Cl | 0.171328 | -1.41855 | -2.04645 |
| H | -2.4942 | -1.74178 | -0.05449 |
| H | 2.718569 | -0.99316 | -1.49243 |
| H | -7.08801 | 1.094287 | 1.144837 |
| H | -4.66793 | 1.307379 | 0.768542 |
| H | -7.01156 | -2.90917 | -0.38391 |
| H | -4.60596 | -2.74144 | -0.77048 |
| H | 4.803083 | 1.456945 | 0.71934 |
| H | 7.248484 | 1.248637 | 0.942442 |
| H | 4.737027 | -2.35173 | -1.35044 |
| H | 7.15859 | -2.52173 | -1.08014 |

**Supplementary table 4. Cartesian coordinates of Ni(II) complex**

| **Atom** | **x** | **y** | **z** |
| --- | --- | --- | --- |
|  |  |  |  |
| C | 5.570116 | -2.16219 | -0.28848 |
| C | 4.281285 | -1.69445 | -0.5005 |
| C | 4.04008 | -0.32158 | -0.57929 |
| C | 5.080385 | 0.597662 | -0.42888 |
| C | 6.377964 | 0.126942 | -0.20306 |
| C | 6.627441 | -1.25355 | -0.14262 |
| O | 7.882648 | -1.69983 | 0.048485 |
| H | 8.084517 | -2.64655 | 0.06393 |
| C | 2.670729 | 0.082469 | -0.84406 |
| N | 1.775847 | 0.128435 | 0.086339 |
| N | 2.096595 | -0.03789 | 1.418043 |
| C | 1.143984 | 0.380404 | 2.373174 |
| N | 1.377431 | 0.233964 | 3.762478 |
| H | 3.009294 | -0.39784 | 1.732618 |
| H | 2.215545 | -0.25493 | 4.104626 |
| H | 0.70931 | 0.620727 | 4.446695 |
| S | -0.19572 | 1.128442 | 1.849324 |
| C | -5.85368 | -2.1373 | -0.82967 |
| C | -4.6115 | -1.65449 | -1.22324 |
| C | -5.94374 | -0.45673 | 0.914194 |
| C | -4.69856 | 0.041562 | 0.511306 |
| C | -4.01742 | -0.575 | -0.54933 |
| C | -6.51284 | -1.55867 | 0.260459 |
| O | -7.66827 | -2.083 | 0.713248 |
| H | -8.0363 | -2.8979 | 0.340575 |
| C | -2.58157 | -0.36993 | -0.7715 |
| N | -1.81793 | 0.637548 | -0.48495 |
| N | -2.22087 | 1.939087 | -0.29806 |
| C | -1.19659 | 2.912023 | -0.52212 |
| N | -1.46164 | 4.303118 | -0.5053 |
| H | -3.20744 | 2.22698 | -0.25687 |
| H | -2.38888 | 4.666275 | -0.25439 |
| H | -0.71415 | 4.969982 | -0.7594 |
| S | 0.296846 | 2.407079 | -0.96767 |
| Ni | -0.02011 | 0.357761 | -0.23935 |
| Cl | -0.30075 | -1.67208 | 0.561969 |
| Cl | 0.09433 | -0.46066 | -2.26729 |
| H | -2.11714 | -1.3003 | -1.05132 |
| H | 2.429772 | 0.279509 | -1.87519 |
| H | -6.43563 | -0.02785 | 1.777252 |
| H | -4.24022 | 0.820341 | 1.104044 |
| H | -6.27151 | -2.99672 | -1.33875 |
| H | -4.08101 | -2.16722 | -2.01862 |
| H | 4.886478 | 1.662842 | -0.48268 |
| H | 7.188719 | 0.833003 | -0.08452 |
| H | 3.465248 | -2.40018 | -0.61925 |
| H | 5.740032 | -3.2296 | -0.24492 |


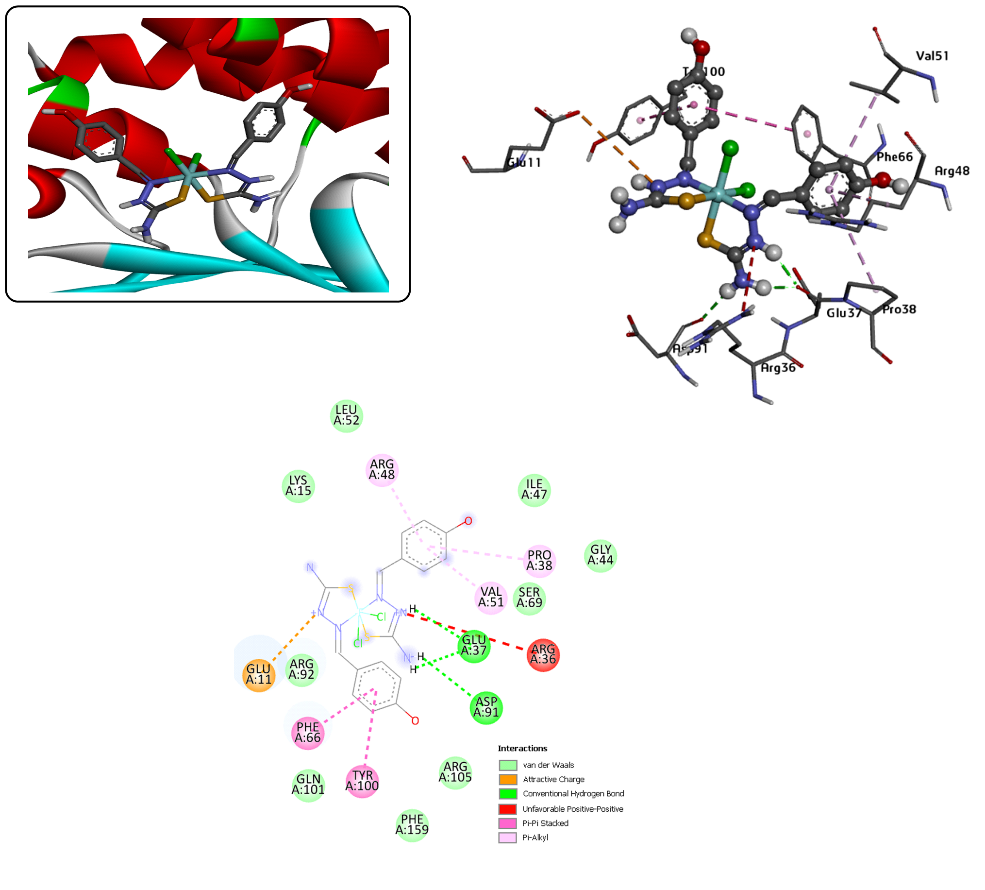


**Supplementary figure 5. Docking score and 3D, 2D interactions of the the synthesized complex [FeL_2_Cl_2_] with target bacterial enzyme 4XWA,**


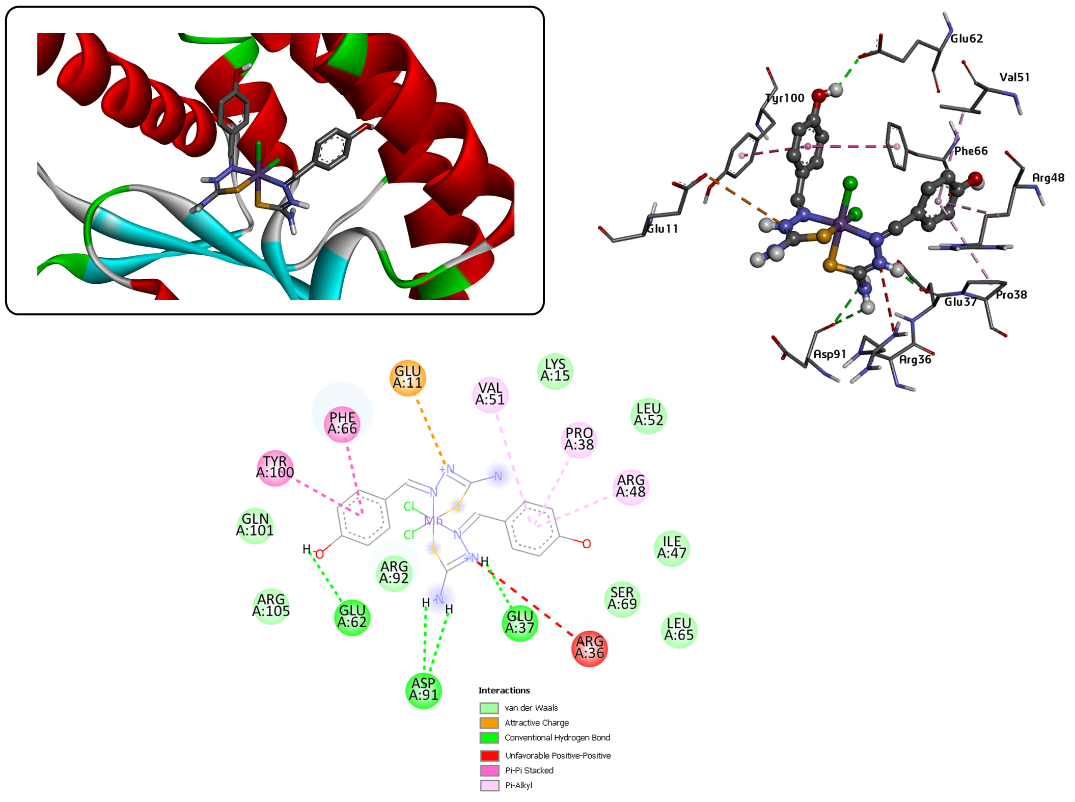


**Supplementary figure 6. Docking score and 3D, 2D interactions of the the synthesized complex [MnL_2_Cl_2_] with target bacterial enzyme 4XWA,**


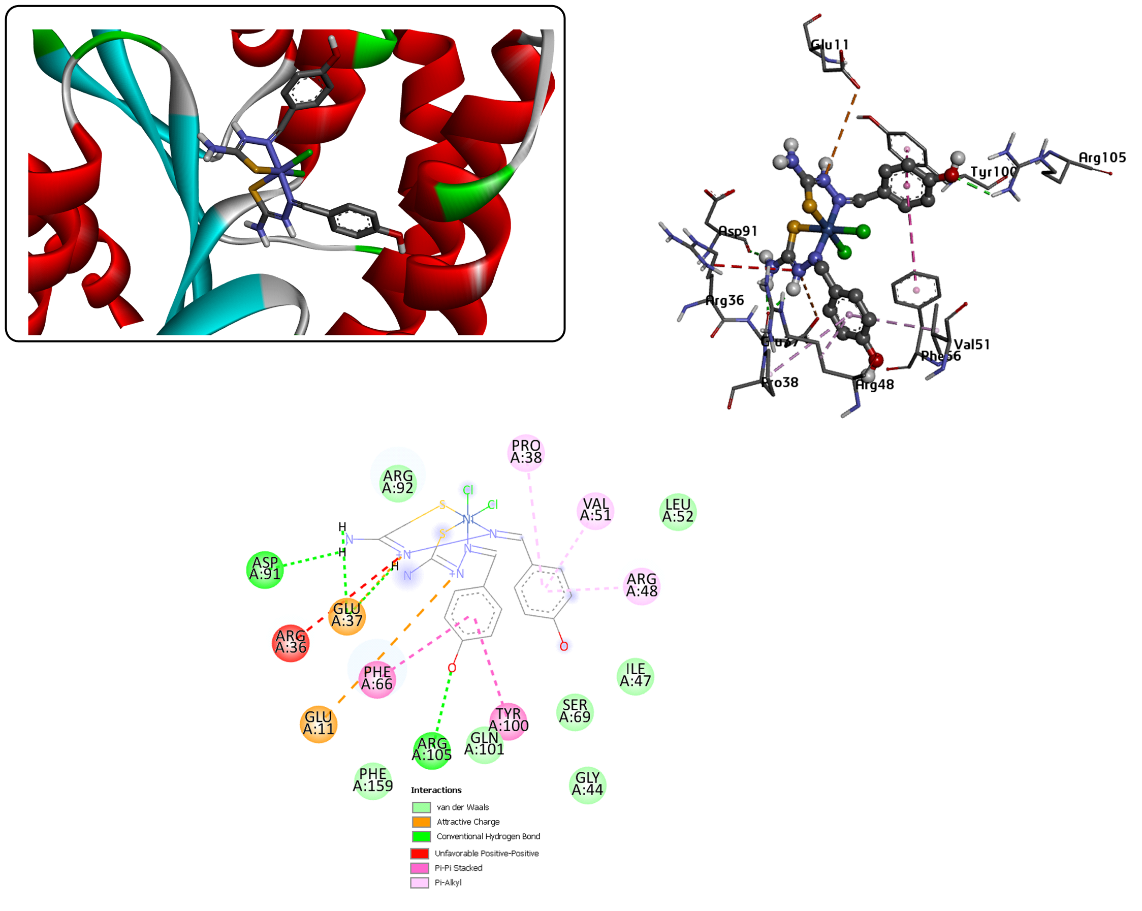


**Supplementary figure 7. Docking score and 3D, 2D interactions of the the synthesized complex [NiL_2_Cl_2_] with target bacterial enzyme 4XWA,**


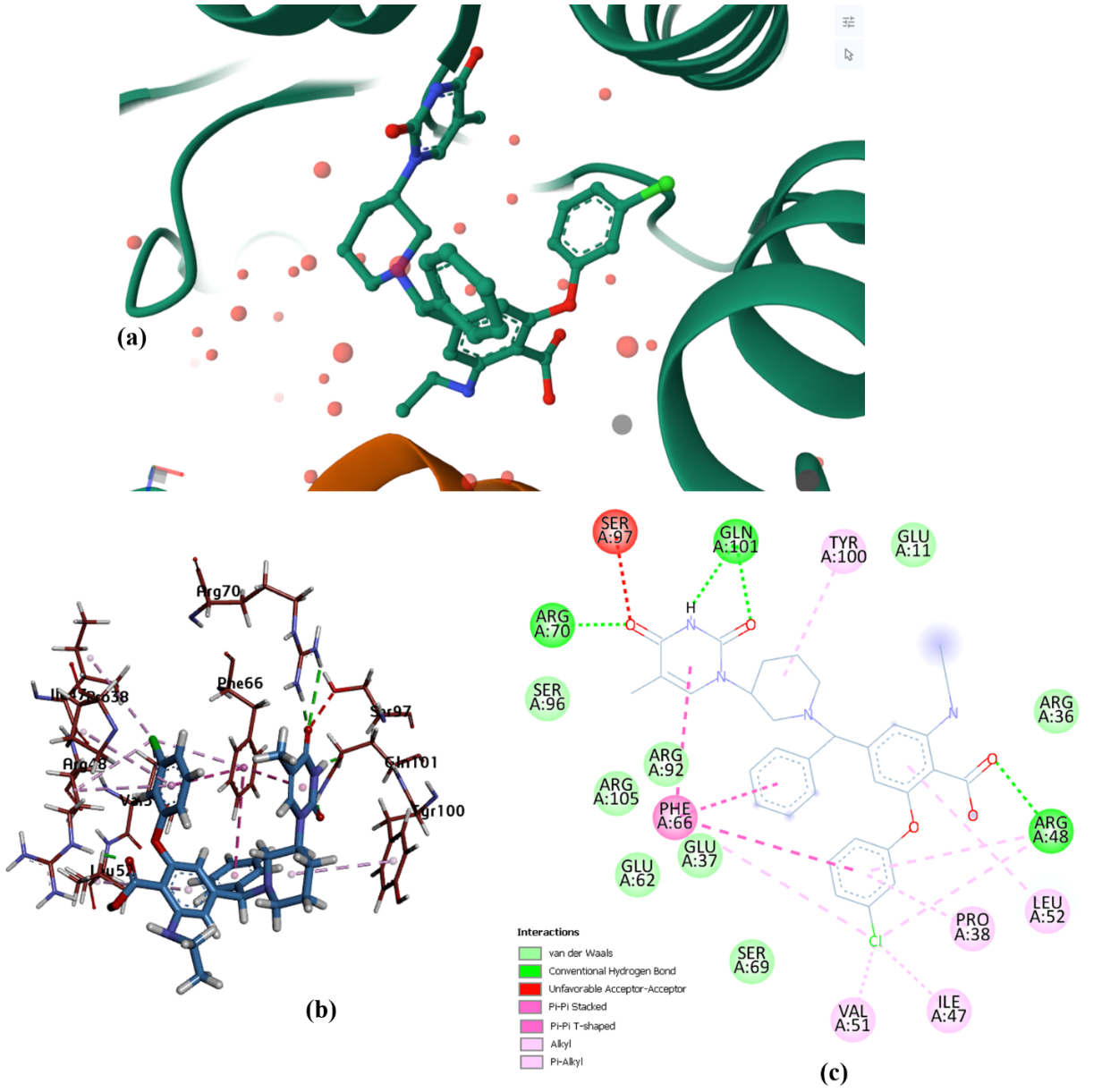


**Supplementary figure 8. Docking Results of the co-crystalized control, (a) crystallographic protein structure including the control, (b) 3D-binding mode of the control in the active stie, (c) 2D-map representing the type of interactions present through control-docking**
